# FOXO1 regulates uterine epithelial integrity and progesterone receptor expression critical for embryo implantation

**DOI:** 10.1371/journal.pgen.1007787

**Published:** 2018-11-19

**Authors:** Yasmin M. Vasquez, Xiaoqiu Wang, Margeaux Wetendorf, Heather L. Franco, Qianxing Mo, Tianyuan Wang, Rainer B. Lanz, Steven L. Young, Bruce A. Lessey, Thomas E. Spencer, John P. Lydon, Francesco J. DeMayo

**Affiliations:** 1 Department of Molecular and Cellular Biology and Center for Reproductive Medicine, Baylor College of Medicine, Houston, TX, United States of America; 2 Reproductive and Developmental Biology Laboratory, National Institute of Environmental Health Sciences, Research Triangle Park, NC, United States of America; 3 Department of Animal Science, North Carolina State University, Raleigh, NC, United States of America; 4 Department of Medicine and Dan L. Duncan Cancer Center, Baylor College of Medicine, Houston, TX, United States of America; 5 Department of Obstetrics and Gynecology, University of North Carolina, Chapel Hill, NC, United States of America; 6 Department of Obstetrics and Gynecology, University of South Carolina School of Medicine, Greenville, SC, United States of America; 7 Division of Animal Science, University of Missouri, Columbia, MO, United States of America; University of Pennsylvania, UNITED STATES

## Abstract

Successful embryo implantation requires a receptive endometrium. Poor uterine receptivity can account for implantation failure in women who experience recurrent pregnancy loss or multiple rounds of unsuccessful *in vitro* fertilization cycles. Here, we demonstrate that the transcription factor Forkhead Box O1 (FOXO1) is a critical regulator of endometrial receptivity *in vivo*. Uterine ablation of *Foxo1* using the progesterone receptor Cre (*Pgr*^*Cre*^*)* mouse model resulted in infertility due to altered epithelial cell polarity and apoptosis, preventing the embryo from penetrating the luminal epithelium. Analysis of the uterine transcriptome after *Foxo1* ablation identified alterations in gene expression for transcripts involved in the activation of cell invasion, molecular transport, apoptosis, β-catenin (CTNNB1) signaling pathway, and an increase in PGR signaling. The increase of PGR signaling was due to PGR expression being retained in the uterine epithelium during the window of receptivity. Constitutive expression of epithelial PGR during this receptive period inhibited expression of FOXO1 in the nucleus of the uterine epithelium. The reciprocal expression of PGR and FOXO1 was conserved in human endometrial samples during the proliferative and secretory phase. This demonstrates that expression of FOXO1 and the loss of PGR during the window of receptivity are interrelated and critical for embryo implantation.

## Author summary

Human fertility is an inefficient process whereby 30% of pregnancies cease before the second trimester. The uterine environment plays a critical role in establishing and maintaining a successful pregnancy. Here, we show that the transcription factor Forkhead Box O1 (FOXO1) increases in the nuclei of endometrial epithelia of both human and mice during the time of embryo implantation, and thus, hypothesize that FOXO1 plays an important role in governing uterine receptivity. To test this hypothesis, we ablated *Foxo1* in the mouse uterus. These mice were infertile due to defective implantation and invasion. The mutant mice demonstrated increased PGR signaling and retained PGR expression in the endometrial epithelia at the window of receptivity. By generating mice with deregulated uterine epithelial *Pgr* expression, we further demonstrated that constitutive expression of epithelial PGR during the receptive period inhibited the expression of FOXO1 in the uterine epithelium. Such reciprocal expression of PGR and FOXO1, similar to a Yin-and-Yang balance, was conserved in human endometrium during the proliferative and secretory phase, suggesting FOXO1 gain and PGR loss in the uterine epithelium is a marker and regulator of endometrial receptivity necessary for the establishment of pregnancy.

## Introduction

The uterine endometrium is receptive to the implanting embryo only during a discrete period in the female reproductive cycle, known as the “window of receptivity” (WOR). During the WOR, the implanting blastocyst and the endometrium engage in intricate and reciprocal physical and metabolic interactions [1]. The synchronous, complementary expression of growth factors, extracellular matrix components, and membrane-bound receptors during this time is critical for anchoring the implanting blastocyst to the apical surface of the luminal epithelium (LE) and guiding the trophoblasts invasion through this barrier [1, 2]. This highly invasive process establishes a fetal-maternal interface for nutrient and gas exchange [3]. The mechanisms by which the trophoblasts breach the LE barrier and progressively displace the underlying decidua are not fully understood, but have been proposed to involve the timely removal of the epithelial barrier by the process of entosis[4–6]. Incomplete or defective implantation is associated with cases of recurrent implantation failure in patients undergoing *in vitro* fertilization and in complications of pregnancy, such as preeclampsia, underscoring the clinical importance for understanding the mechanisms involved in establishing a receptive endometrium [7–10].

The WOR is regulated at the level of gene expression by the ovarian hormones estrogen and progesterone acting via their cognate nuclear receptors, the estrogen receptor (ESR1) and the progesterone receptor (PGR). These receptors work in conjunction with a number of transcription factors and coregulators to regulate uterine physiology [11, 12]. One of the transcription factors which co-regulates PGR action is the Forkhead Box O1 (FOXO1). FOXO1 is a member of the Forkhead domain family. FOXO1 has been observed to interact with PGR in decidualized human endometrial stromal cells *in vitro* to regulate target gene expression that controls cell proliferation and epithelioid differentiation [13]. FOXO1, like other members of the family, is involved in the regulation of cell cycle, apoptosis, and modulation of metabolic flux. While expression of FOXO1 is known to be induced and required in the decidualization of human endometrial stromal cells in tissue culture settings [14], the *in vivo* role of FOXO1 in the uterus prior to implantation has not been investigated. The aims of this study were to define the expression pattern of FOXO1 in the human and murine endometrium, to determine the functional roles of FOXO1 in steroid signaling, and to determine the requirement of FOXO1 in female fertility. We report that FOXO1 is localized in the nucleus of the luminal (LE) and glandular (GE) epithelium of the endometrium during the WOR in humans and mice and in the cytoplasm of the murine decidua as pregnancy processes. Since the whole body ablation of FOXO1 results in embryonic lethality [15], we used the Pgr^Cre^ mouse model to conditionally ablate Foxo1 in PGR expressing cells. The Pgr^Cre^ edits genes in the pituitary, preovulatory ovary granulosa cells, mammary gland and all compartments of the mouse uterus [16, 17]. The conditional ablation of *Foxo1* using the Pgr^Cre^ resulted in infertility due to disrupted implantation of the blastocyst. Surprisingly, the post implantation decidual process was not severely impacted. Analysis of the FOXO1-dependent transcriptome at day 4.5 of pseudopregnancy (PPD 4.5) identified several important pathways involved in the fetal-maternal crosstalk regulated by FOXO1. The processes identified correspond to those that allow the removal of the epithelial barrier for trophoblastic invasion. One of the processes altered was the expression of PGR in the uterine epithelium during the WOR. We then demonstrated that expression of FOXO1 and loss of PGR are interrelated and a can serve as a marker for the WOR in mice and humans. [17][15]

## Results

### Endometrial expression of FOXO1 during the preimplantation period

**Fig 1:**
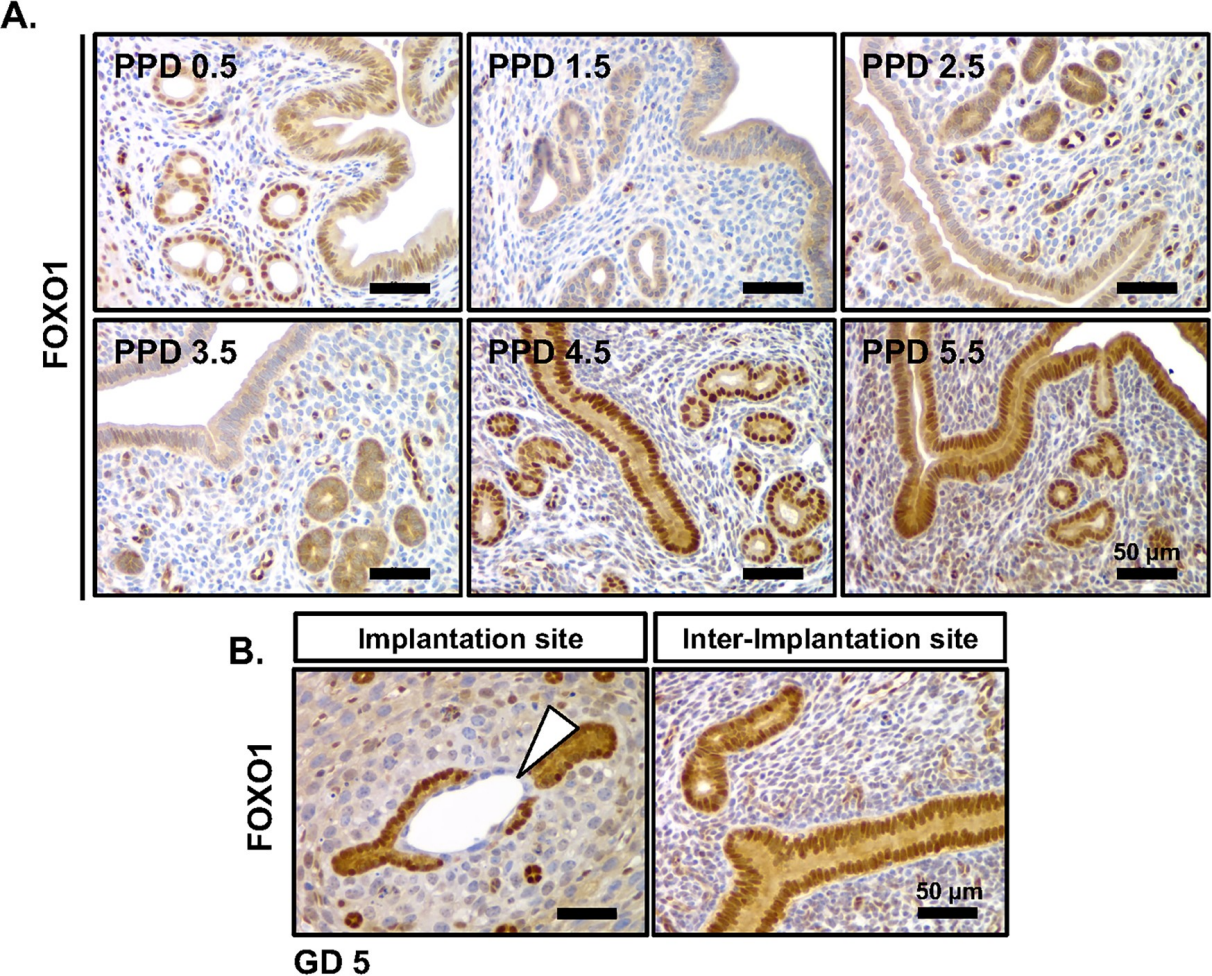
Endometrial expression of FOXO1 during the preimplantation period in the uteri of pseudopregnant mice. **(A)** Immunohistochemical staining for FOXO1 in endometrial cross sections of pseudopregnant (PP) mice (*n* = 3). **(B)** Immunohistochemical staining FOXO1 in sagittal sections of the implantation site (IS) and inter-implantation site (I-IS) in the afternoon of the window of receptivity (gestational day 5, GD 5)(*n* = 3). Blastocyst indicated by the white triangle. Scale bar, 50 μm.

Expression of FOXO1 was evaluated in the murine endometrium during the peri-implantation period of pregnancy. Immunohistochemical analysis was conducted on the uteri of pseudopregnancy day (PPD) 0.5 to day 5.5 C57BL/6J female mice. At PPD 0.5, FOXO1 protein was detected in the nucleus of the GE and LE. From PPD 1.5 to 3.5, FOXO1 protein was detected at low levels in the cytoplasm of the LE and GE. At PPD 4.5, FOXO1 protein was localized to the nucleus in both the GE and LE. At PPD 5.5 GE and LE FOXO1 protein was retained in the nucleus and weak cytoplasmic expression was evident (**Fig 1A).**

The expression of FOXO1 was next examined during pregnancy in the mouse. Previous *in vitro* studies [14, 18] spurred the expectation that FOXO1 expression would be induced in the stromal compartment during decidualization. [19] FOXO1 expression in the uteri at gestational day (GD) 5 revealed a similar expression pattern as that seen at PPD 5.5. The nuclei of the LE and GE adjacent to the implanting embryo, as well as, in the inter-implantation site (I-IS) were immune-positive for FOXO1. Expression of FOXO1 is maintained around the implanting embryo where the epithelium has not been removed by embryo implantation. **(Fig 1B, arrow)**. While the staining for FOXO1 was negative in both the nucleus and cytoplasm of the primary decidual zone (PDZ), the cytoplasm of the stroma surrounding the PDZ was faintly immune-positive for FOXO1 **(Fig 1B)**. Furthermore, FOXO1-staining localized to the cytoplasm of decidual cells, while blood vessels displayed nuclear immunostaining for FOXO1 (**Fig 1B**). The endometrial compartment-specific and temporal subcellular localization of FOXO1 in the pregnant murine endometrium suggests a potential role of FOXO1 during the WOR.

### Endometrial ablation of Foxo1

**Fig 2:**
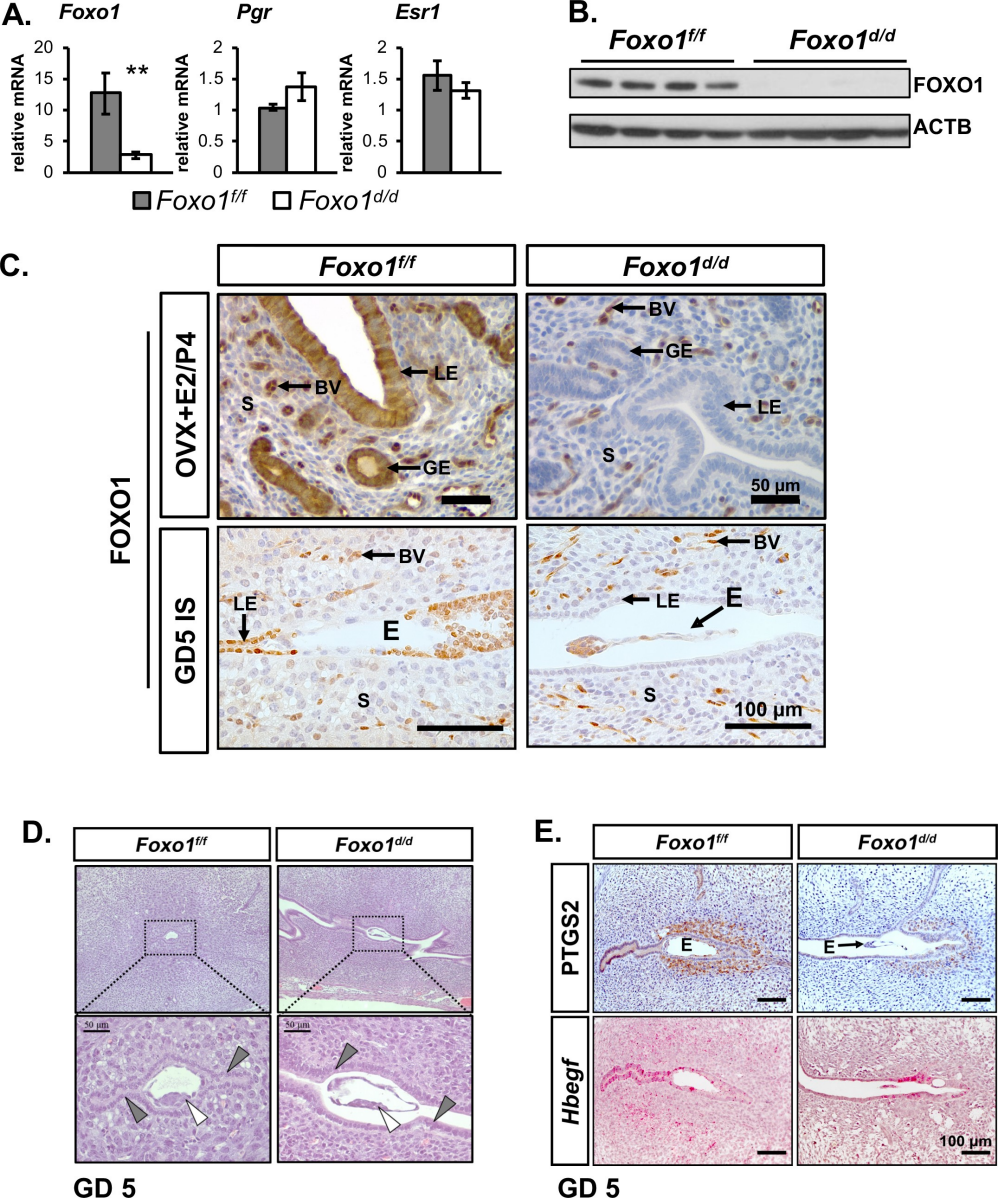
Ablation of *Foxo1* in the endometrium results in infertility. **(A-B)** Generation of a *Foxo1* conditional knockout (*Foxo1*^*d/d*^) using the *Pgr*^*Cre*^ mouse model in adult ovariectomized females treated with estrogen and progesterone to mimic day 3.5 of pregnancy. **(A)** Ablation of *Foxo1* message was determined by qRT-PCR. Ablation of *Foxo1* does not affect the expression of the progesterone (*Pgr*) and estrogen receptor (*Esr1*) genes. Data are presented as means ± SEM, *n* = 6. ** *P*<0.01. **(B)** Western blot analysis *of Foxo1* ablation with β-actin (ACTB) as protein loading control. **(C)** Immunohistochemistry with a FOXO1-specific antibody of uterine cross sections in estrogen (E2) and progesterone (P4)-primed ovariectomized (OVX) mice (OVX+E2/P4) and at GD 5 (*n* = 3). Arrows indicate endometrial compartments as follows: LE, luminal epithelium; GE, glandular epithelium; BV, blood vessel; S, stroma; and E, embryo. IS, implantation site. Scale bars: 50 μm for top panel; 100 μm for bottom panel. **(D)** Intact females at 6 weeks of age were mated with fertile males. Presence of vaginal plug indicated postcoital day 0.5 (GD 0.5). Eosin and hematoxylin staining was performed on serial sagittal sections of whole uteri dissected at GD 5 (*n* = 3). Attachment sites in *Foxo1*^*f/f*^ and *Foxo1*^*d/*d^ are shown in low magnification and high magnification of dotted box area. Inner cell mass of blastocyst indicated by white triangle. LE cells indicated by grey triangles. Scale bar, 50 μm. **(E)** Immunohistochemical staining for PTGS1 and RNAscope *in situ* hybridization for *Hbegf* on transverse serial sections at GD 5 (*n* = 4). E, embryo. Scale bar, 100 μm.

The impact of *Foxo1* ablation on female mouse fertility was determined by the conditional ablation of *Foxo1* in the mouse uterus. Generation of the conditional allele targeting the second major coding exon of *Foxo1* (encoding the C-terminal half of the full-length protein) was previously described. [20] Floxed *Foxo1* (*Foxo1*^*f/f*^) mice were crossed with the *Pgr*^*Cre*^ mouse model [16] to generate *Pgr*^*Cre*^ *Foxo1*^*f/f*^ (*Foxo1*^*d/d*^) animals. Validation of the tissue specific ablation of Foxo1 was performed on ovariectomized (OVX) females primed with estrogen (E2) and progesterone (P4) to mimic day 3.5 of pregnancy (OVX+E2/P4). Quantitative real time qPCR shows significantly attenuated levels of *Foxo1* transcripts in the uteri of *Foxo1*^*d/d*^ females when compared to the *Foxo1*^*f/f*^ females (**Fig 2A).** Notably, the *Foxo1* knockout appeared to have no effect on the expression of the genes encoding for the progesterone receptor (*Pgr*) and estrogen receptor alpha (*Esr1*) at this time point. Evidence of ablation was also confirmed by Western blot analysis of FOXO1 protein levels (**Fig 2B).** This hormonal regimen was favored over a natural mating time point to circumvent any variation resulting from differences in mating time, or the contribution of embryonically-derived tissue during the molecular analysis. Immunostaining for FOXO1 was also conducted at GD 5 in the uteri of *Foxo1*^*f/f*^ and *Foxo1*^*d/d*^ females mice. This staining showed that while FOXO1 immunoreactivity was present in the LE and GE and uterine blood vessels, of control *Foxo1*^*f/f*^ mice while FOXO1 was only detected in the uterine blood vessels of *Foxo1*^*d/d*^ females, demonstrating that in the uterus, ablation was specific to the epithelial and stromal compartments (**Fig 2C).**

### Abnormal embryo Implantation and blastocyst attachment

The impact of *Foxo1* ablation of female fertility was evaluated by conducting a 6-month breeding trial during which the control *Foxo1*^*f/f*^ and knockout *Foxo1*^*d/d*^ females were mated with wild-type male mice. During the breeding trial, all females were observed to mate normally. *Foxo1*^*f/f*^ female mice produced an average of 6 +/- 0.52 pups in an average of 8.97 +/- 0.37 litters while *Foxo1*^*d/d*^ females were unable to produce a single litter.

In order to determine the cause of infertility, control *Foxo1*^*f/f*^ and knockout *Foxo1*^*d/d*^ females were mated to intact males and sacrificed at daily intervals from GD 6.5 to 9.5. This analysis showed that there was no impact on the number of implantation sites, IS, (**S1A Fig)**; growth of the IS in diameter was quantified from mesometrium (M) to antimesometrium (AM) and determined that at GD 6.5 and 7.5, the average IS size in the knockout females was slightly but significantly smaller than the IS of control females. The diameter of the ISs were not significantly different at GD 8.5 or 9.5 (**S1B Fig)**. Histological analysis of the implantation sites was conducted to determine the impact of *Foxo1* ablation on embryo development. The embryo proper and placenta were normal in all *Foxo1*^*f/f*^ pregnancies as judged by eosin and hematoxylin staining of the mid-section of IS (**S1C Fig**). In the knockout mouse, no embryo could be detected in the IS at any stage evaluated and consequently it lacked a developing placenta. Examination of all the ISs collected at GD 9.5 revealed that they were in various stages of decidual regression, indicative of failed pregnancy and resorption (**S1C Fig)**. These findings demonstrated that embryos made contact with the uterine epithelium and was able to induce a decidual reaction. However, subsequent invasion development of the embryo was impaired. We next investigated the ability of the blastocyst to penetrate the LE.

With the observation that FOXO1 exhibits nuclear localization in the epithelium on the day of implantation and the failure of development of the post implantation, we next evaluated the functional requirement of *Foxo1* gene attachment and invasion of the blastocyst to the uterine epithelium. It is known that initial apposition of the blastocyst to the surface of the LE is characterized by a weak association that is followed by a stronger attachment interaction at GD 4.5. To evaluate the role of the *Foxo1* gene in implantation, females were mated with fertile males and IS were examined at GD 5 (afternoon of GD 4.5). At this time, all the blastocysts in *Foxo1*^*f/f*^ control females were found to be closely opposed to the LE (**Fig 2D**). Among the 6 female GD 5 *Foxo1*^*d/d*^, only 13% (3/23) were found to be closely opposed to the LE. The rest were either loosely opposed, 39% (9/23) or floating in the luminal fluid, 47% (11/23). The LE in *Foxo1*^*d/d*^ mouse ISs appeared incompletely enclosed upon the blastocyst, suggesting an aberrant fluid resorption required for luminal closure, a process that is critical for successful murine implantation [21] (**Fig 2D).** The uterine receptivity markers Hbegf and PTGS2 were examined by RNAscope in situ hybridization and immunohistochemistry, respectively, at GD 5 to determine if loss of Foxo1 resulted in the altered epithelial expression of HB-EGF or stromal expression of PTGS2. The expression of Hbegf was not significantly disrupted in the uterine epithelium of the *Foxo1*^*d/d*^ mouse, while PTGS2 was reduced in the uterine stroma of the *Foxo1*^*d/d*^ mouse **(Fig 2E)**. This indicates that *Foxo1* has a different impact on the mouse uterine receptivity markers not affecting HB-EGF expression that is involved in embryo attachment but affecting stromal PTGS2, which is a marker of embryo induction of the stroma decidualization.

**Fig 3:**
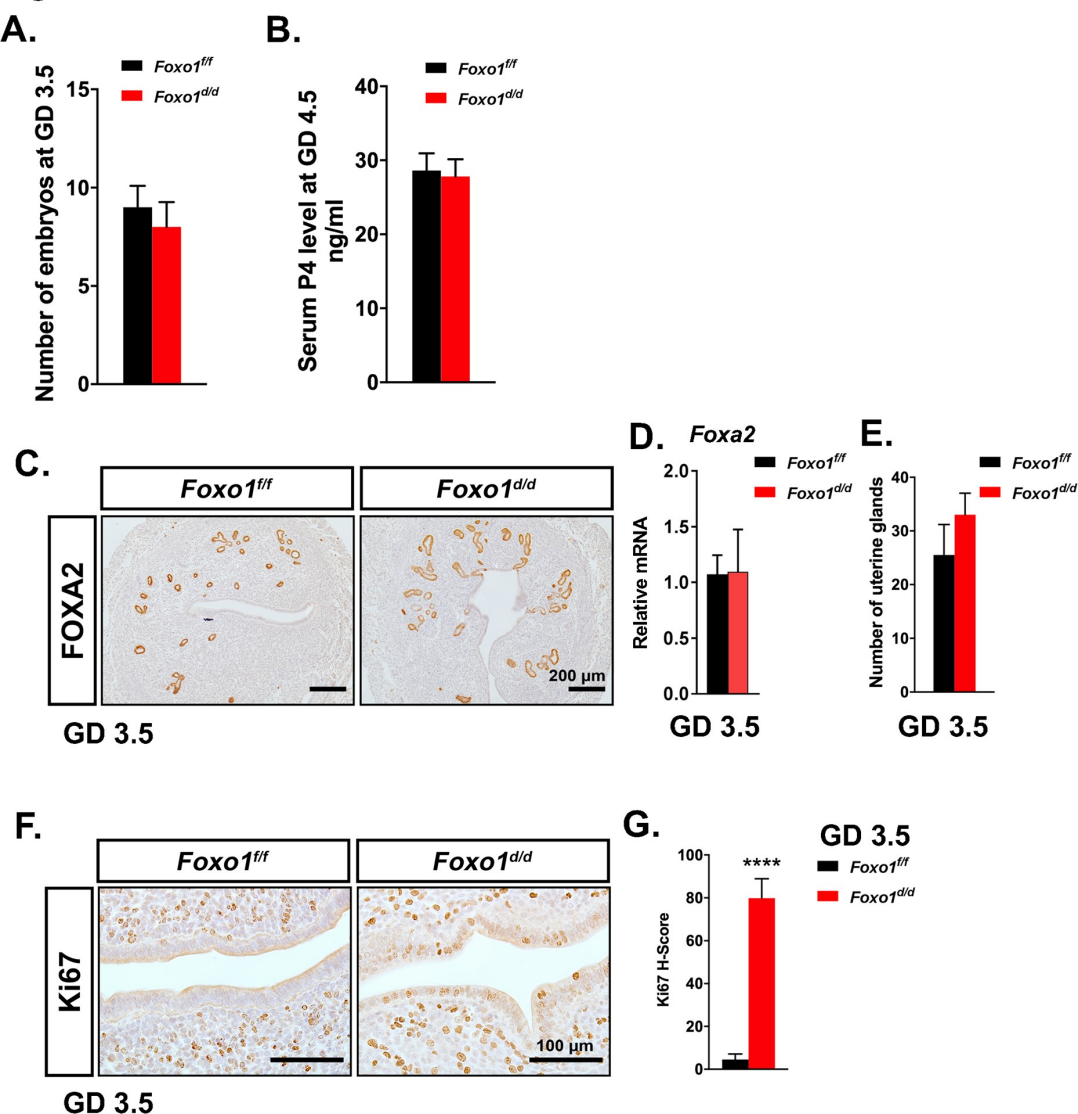
Ablation of *Foxo1* causes continues proliferation in uterine epithelia independent of ovarian function and adenogenesis. **(A)** The number of embryos flushed from *Foxo1*^*f/f*^ and *Foxo1*^*d/d*^ uteri at GD 3.5 (*n* = 5). **(B)** Serum P4 level from *Foxo1*^*f/f*^ and *Foxo1*^*d/d*^ mice at GD 3.5 (*n* = 5). **(C)** Immunohistochemical staining of FOXA2 in GD 3.5 uteri from *Foxo1*^*f/f*^ and *Foxo1*^*d/d*^ mice (*n* = 4). Scale bar, 200 μm. **(D)** Quantification of *Foxa2* gene in GD 3.5 uteri from *Foxo1*^*f/f*^ and *Foxo1*^*d/d*^ mice (*n* = 6). **(E)** The number of uterine glands from *Foxo1*^*f/f*^ and *Foxo1*^*d/d*^ mice (*n* = 4) at GD 3.5. **(F)** Immunohistochemical staining of Ki67 in GD 3.5 uteri from *Foxo1*^*f/f*^ and *Foxo1*^*d/d*^ mice (*n* = 4). Scale bar, 100 μm. **(G)** H-score quantification of Ki67 in endometrial section of *Foxo1*^*f/f*^ and *Foxo1*^*d/d*^ mice (n = 3) at GD 3.5. Data are presented as means ± SEM. ****, *P*<0.0001.

Since the PGR^Cre^ model ablates genes in the pituitary, the preovulatory granulosa cells of the ovary, and the uterus starting at birth, we next determined if the resulting phenotype was due to an ovarian defect. The involvement of the pituitary-ovary axis in the sterility phenotype *Foxo1*^*d/d*^ mice was assayed by determining if the ovary could ovulate and produce normal embryos and maintain serum progesterone levels during the preimplantation period. As seen in **Fig 3A** and **3B** the number of blastocysts collected at GD 3.5 and serum progesterone levels at GD 4.5 shows no significant difference between the *Foxo1*^*f/f*^ and *Foxo1*^*d/d*^ female mice, respectively. This indicates that the ovary was capable of ovulating and producing progesterone to maintain pregnancy. In order to determine if there was a developmental defect in the uterus, immunohistochemical analysis of the uterine gland specific marker FOXA2 was utilized to investigate uterine morphology and uterine gland gene expression. This analysis demonstrated that there was no obvious morphological abnormality in the uterus or uterine glands and that the uterine glands expressed FOXA2 (**Fig 3C)**. [22] *Foxa2* expression was then quantified by real time quantitative PCR and no difference in the level of *Foxa2* was observed between the *Foxo1*^*f/f*^ and *Foxo1*^*d/d*^ female mice **(Fig 3D)**. The number of uterine glands was counted and there was no difference in the number of uterine glands between the *Foxo1*^*f/f*^ and *Foxo1*^*d/d*^ female mice **(Fig 3E).** Therefore, ablation of Foxo1 caused no obvious abnormality in uterine structure or gland development.

Events in the preimplantation period are critical for the regulation of the WOR. One of the critical events in the preimplantation period is the cessation of uterine epithelial proliferation and the expression of the cytokine leukemia inhibitory factor, LIF. LIF is expressed on GD 3.5 from the uterine glands and is necessary for embryo attachment and implantation [23]. Analysis of uterine epithelial cell proliferation was assayed by immunohistochemical analysis for Ki67. As shown in **Fig 3F**, the *Foxo1*^*f/f*^ showed Ki67 staining in the stroma compartment with the epithelium devoid of Ki67 immunoreactivity. The *Foxo1*^*d/d*^ uteri showed Ki67 positive staining in both the epithelial and stromal compartments of the uterus at GD3.5 **(Fig 3F).** Quantification of the Ki67 positive epithelial cells in the uteri of the Foxo*1*^*f/f*^ and *Foxo1*^*d/d*^ mice showed a significant increase in the number of Ki67 immunopositive cells in the epithelium of the *Foxo1*^*d/d*^ mouse uteri. **(Fig 3G)**. This analysis demonstrates that loss of FOXO1 alters the control of uterine epithelial cell proliferation in the preimplantation period.

Analysis of the expression of *Lif* mRNA was conducted by real time quantitative PCR at GD 3.5 and 4.5. The expression of *Lif* was significantly lower at GD 3.5 in the *Foxo1*^*d/d*^ and significantly increased in the *Foxo1*^*d/d*^ at GD 4.5 (**S2A Fig)**. This indicates that the preimplantation induction of *Lif* was delayed. In order to determine if the infertility phenotype could be explained by a delay in *Lif* induction, a rescue of the implantation was attempted using recombinant LIF (rLIF). *Foxo1*^*f/f*^ and *Foxo1*^*d/d*^ mice were mated to an intact male mouse using a previously published protocol [24]. *Pgr*^*Cre*^ *Foxa2*^*f/f*^ (*Foxa2*^*d/d*^) female mice served as a positive control in this experiment. *Foxa2*^*d/d*^ do not have uterine glands and do not support embryo implantation due to lack of production of LIF. The implantation defect in these mice can be rescued by rLIF [24]. In this experiment, rLIF did not rescue the embryo attachment phenotype of the *Foxo1*^*d/d*^ but was able to rescue the embryo implantation defect in *Foxa2* ^*d/d*^ (**S2B Fig).** Therefore, ablation of *Foxo1* causes altered uterine epithelial cell proliferation and a delayed induction of LIF expression. However, the delayed in LIF expression was not the sole cause of the embryo implantation defect.

### Role of FOXO1 in a murine model of decidualization

Previous evidence from *in vitro* decidualization of human endometrial stromal cells identified FOXO1 as a transcriptional co-regulator of PGR transactivation, the latter a requirement for decidualization [13, 14]. To evaluate the role of FOXO1 in decidualization *in vivo*, we implemented a well-defined strategy for artificial induction of the decidual response [25]. Deciduogenic stimulus from instillation of sesame oil to the right uterine horn resulted in a robust increase of biomass of the stimulated horns 2 (DD 2) and 5 (DD 5) days after stimulus delivery (**S3A Fig)**. Quantification of the decidual/control weight ratio indicated that at DD 2, the *Foxo1*^*f/f*^ control and *Foxo1*^*d/d*^ knockout mouse had comparable biomass. However, the increase in biomass of the control horn at DD 5 of the artificial decidual regimen was significantly attenuated in the *Foxo1*^*d/d*^ females (**S3B Fig)**. Immunohistochemical staining with a FOXO1 antibody was performed to assess the endometrial architecture of the decidual horn of both genotypes at DD 2 (**S3C Fig)** and DD5 (**S3D Fig)**. At DD 2 the transformation of stromal cells to rounded decidual cells is observed to initiate in the antimesometrial pole of the stimulated horns. The LE appeared to have completely disintegrated in the *Foxo1*^*f/f*^ decidual horn (**S3C Fig**). LE cells positive for FOXO1 were observed only in the antimesometrial pole where the luminal space has been enclosed. In the *Foxo1*^*d/d*^ decidual horn, the antimesometrial pole appears to have initialized decidual reaction but the lumen is not fully enclosed. (**S3C Fig)**. At DD 5 in the *Foxo1*^*f/f*^ uteri, a region morphologically similar to the primary decidual zone (PDZ) seen in natural pregnancies was visible, but staining for FOXO1 in this region, indicated by the grey triangle, was absent (**S3D Fig)**. The decidual area surrounding the PDZ, indicated by the black triangle, had FOXO1 staining in the cytoplasm. Notably, the LE in the *Foxo1*^*f/f*^ decidual horn appeared to be disintegrating. The remaining LE cells showed significant nuclear staining for FOXO1. At the histological level, the *Foxo1*^*d/d*^ decidual reaction on DD 5 did not appear abnormal (**S3D Fig)**. However, the LE remained intact in the decidual horn of *Foxo1*^*d/d*^ uteri at DD5 (**S3D Fig)**. Analysis of the expressions of decidual marker genes, *Bmp2* and *Wnt4* at DD2 showed no significant difference in the expression of these markers at DD2 (**S3E Fig)**.

### Ablation of Foxo1 results in retained cell polarity of murine uterine epithelium

**Fig 4:**
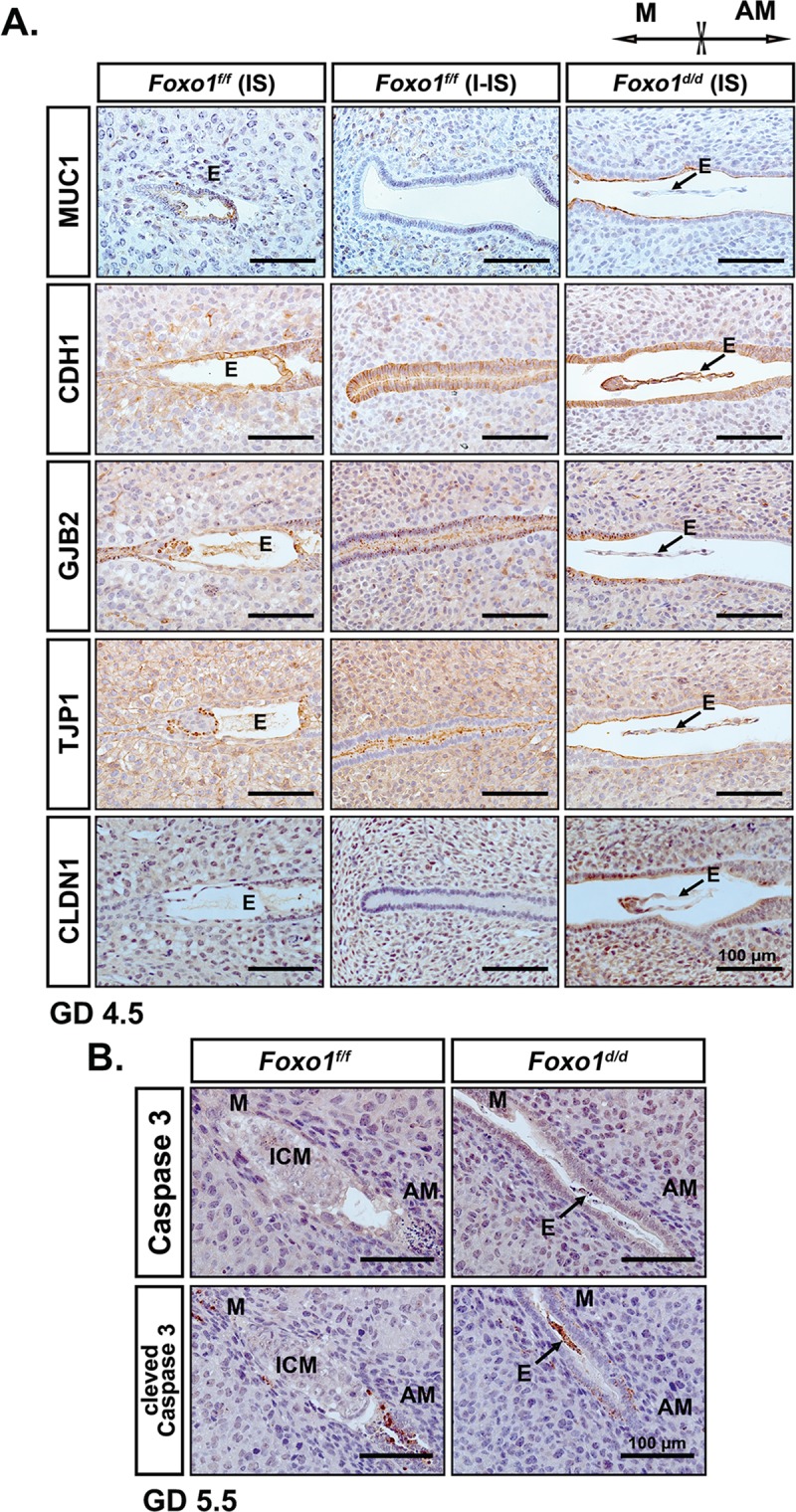
Ablation of *Foxo1* retains the polarity of uterine epithelium in GD 4.5 pregnant mice. (**A**) Immunohistochemical staining for Mucin-1 (MUC1), E-cadherin (CDH1), Connexin 26 (GJB2), ZO-1 (TJP1) and Claudin 1 (CLDN1) in the cross sections of both *Foxo1*^*f/f*^ and *Foxo1*^*d/d*^ murine uteri at GD 4.5. (**B**) Immunohistochemical staining for Caspase 3 and cleaved-Caspases 3 in the cross sections of *Foxo1*^*f/f*^ and *Foxo1*^*d/d*^ murine uteri at GD 5.5. M, mesometrial pole; AM, antimesometrial pole; E, embryo; ICM, inner cell mass; IS, implantation site; I-IS, inter-implantation site. Scale bar, 100 μm.

During the establishment of implantation, the LE shows a decrease in the expression of MUC1 and changes in cell polarity. This allows trophoblasts to attach to the luminal epithelium and initiate epithelial disintegration at GD 4.5 which is followed by apoptosis in the anti-mesometrial (AM) extension of LE at GD 5.5 [5]. Thus, immunohistochemical analyses were conducted on uteri at GD 4.5 and GD 5.5 to determine whether FOXO1 is required for such changes in MUC 1 expression and uterine cell polarity during the WOR and the eventual LE to support embryo implantation (**Fig 4A and S4 Fig**). As shown in **S4B Fig,** the orientation of the embryo implantation with respect to the mesometrial-antimesometrial axis of the embryo was not altered. At GD 4.5 the LE at IS of *Foxo1*^*d/d*^ uteri remained intact and the nucleus was located toward the center of the LE cell (**Fig 4A**). In contrast, the LE at IS of *Foxo1*^*f/f*^ uteri were disintegrating (**Fig 4A**), which is consistent with the observation in the *Foxo1*^*f/f*^ decidual horn at DD5. The LE at I-IS of *Foxo1*^*f/f*^ uteri were also intact. However, its nucleus was located toward the basal membrane of the LE cell (**Fig 4A**). To further analyze apico-basal polarity in LE, IHC localization of Mucin-1 (MUC1; apical surface of LE) and E-cadherin (CDH1; apical-basal-lateral membranes of LE) was performed. The expression of both proteins remained high throughout the LE of *Foxo1*^*d/d*^ uteri at GD 4.5 as compared with that of *Foxo1*^*f/f*^ uteri (**Fig 4A**). Junction proteins, such as Connexin 26 (GJB2) and ZO-1 (TJP1) were absent in the utero-embryonic interface of *Foxo1*^*d/d*^ mice at GD 4.5 while both proteins were readily detected in the IS of *Foxo1*^*f/f*^ uterine LE (**Fig 4A**). GJB2 and TJP1 were positive in the stromal cells of both *Foxo1*^*f/f*^ and *Foxo1*^*d/d*^ uteri. Meanwhile, Claudin 1 (CLDN1) was express in both uterine stromal and epithelial compartments in *Foxo1*^*d/d*^ mice while the *Foxo1*^*f/f*^ mouse uteri only showed expression in the stroma cells at GD 4.5 (**Fig 4A**). The changes at GD 4.5 were also observed at GD 5.5 (**S4A Fig**). In order to determine if these changes in cell polarity markers impacted the loss of the uterine epithelium during implantation, total and cleaved Caspase-3 (apoptosis marker) were evaluated. Deletion of *Foxo1* in uteri dampened the activation of Caspase-3 in the AM extension of LE at GD 5.5, leading to dying embryos that showed high levels of cleaved Caspase-3 (**Fig 4B**). This analysis demonstrates that FOXO1 is critical for the changes in epithelial polarity at receptivity required for embryo invasion.

### RNA-seq analysis of the FOXO1 endometrial gene signature

**Fig 5:**
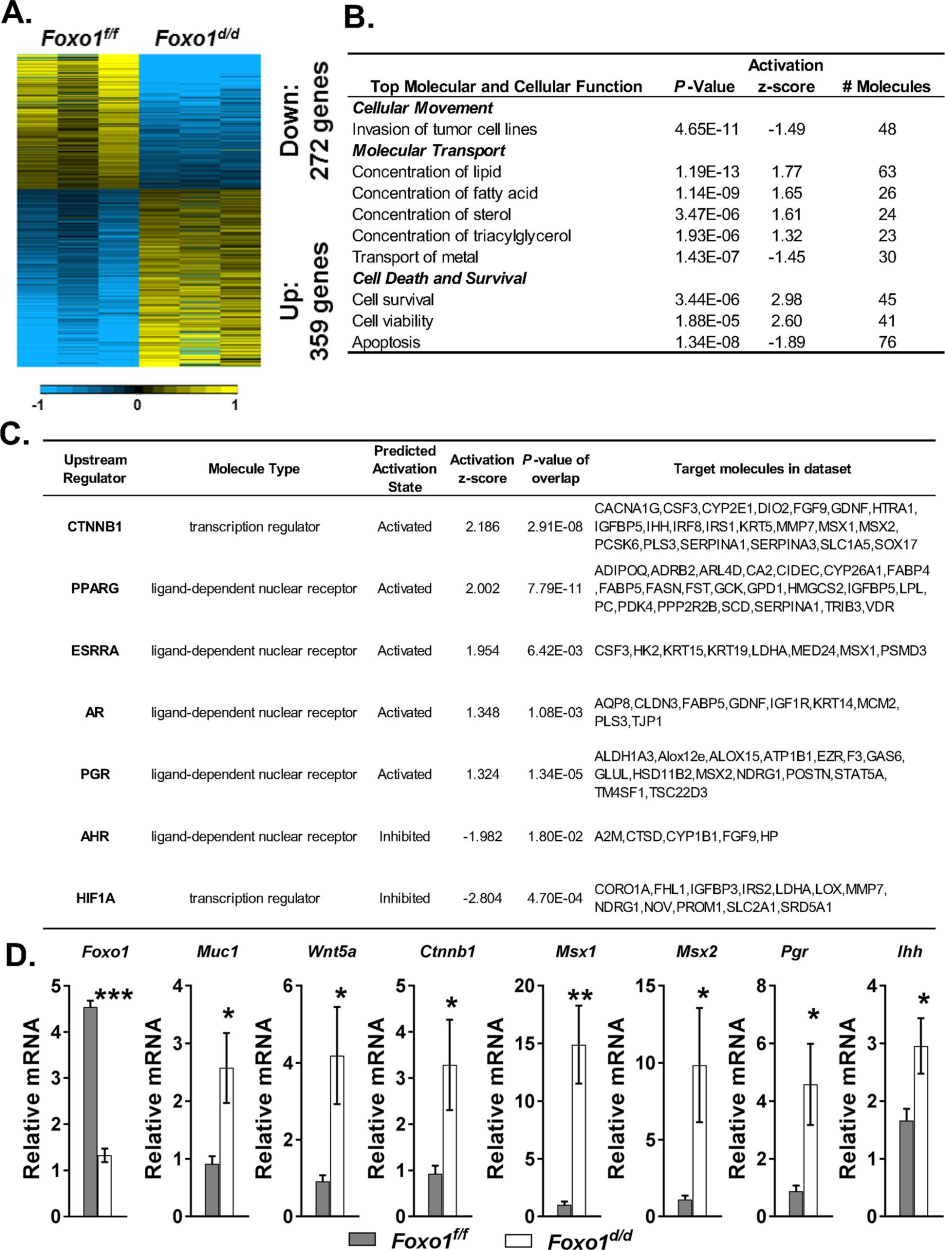
FOXO1-dependent regulation of uterine genes. **(A)** Heat map representation of gene expression levels determined by RNA-seq in *Foxo1*^*f/f*^ and *Foxo1*^*d/d*^ uteri at day 4.5 of pseudopregnancy (PPD 4.5). Differential gene expression analysis identified 631 uterine genes significantly regulated in the *Foxo1*^*d/d*^ mice relative to *Foxo1*^*f/f*^. **(B)** Enrichment of functional annotation and **(C)** activities of upstream regulators in differentially expressed genes between *Foxo1*^*f/f*^ and *Foxo1*^*d/d*^ mouse uteri at PPD 4.5 by IPA analysis. **(D)** Independent samples collected for qRT-PCR validation of targets of interests in the ontology analysis of the FOXO1 gene signature. Data are presented as means ± SEM. *n* = 5. *, *P<*0.05; **, *P<*0.01; and ***, *P*<0.001.

In order to identify the underling molecular mechanism for the recurrent implantation failure phenotype observed in the *Foxo1*^*d/d*^ mice, we performed RNA-seq analysis of gene expression on control and knockout females at day 4.5 of pseudopregnancy (PPD 4.5). Sequencing was performed for each genotype using pooled RNA from three different pools of uteri from two independent mice. This analysis revealed that 631 genes exhibited differential expression between genotypes (*Foxo1*^*d/d*^ vs *Foxo1*^*f/f*^, **S1 Table)**, in which 272 genes were down regulated and 359 genes were upregulated (**Fig 5A).** Functional annotation, upstream regulators, as well as, canonical pathways were analyzed by Ingenuity Pathway Analysis software (IPA, www.ingenuity.com) in order to get an understanding of the underlying pathways responsible for the infertility phenotype. The top 3 molecular and cellular functions associated with *Foxo1* ablation in the uterus were 1) cell invasion, 2) molecular transport, and 3) cell death and survival (**Fig 5B)**. The entire list of functional annotations can be found as **S2 Table**. Furthermore, 34 Ingenuity Canonical Pathways were also affected by uterine *Foxo1* ablation (**S3 Table**), including activated signaling pathways of iNOS [26], BMP [27] and IL-6 [28], as well as, inhibited signaling pathways of p53 [29] and PI3K/AKT [30]. All these pathways have been shown to impact implantation [2, 21, 26–31]. IPA upstream regulator analysis identified several potential upstream molecular factors involved in the *Foxo1d/d* implantation defect. Among these, CTNNB1 (β-Catenin), PPARG, ESRRA, androgen receptor (AR) and PGR -regulated pathways were predicted to be activated, whereas AHR and HIF1A activities were predicted to be inhibited in the *Foxo1d/d* uteri (**Fig 5C)**. The aberrant expression of some of these genes in the murine uterus was validated by qRT-PCR, including *Foxo1*, *Pgr*, *Ihh*, *Lif*, *Msx1*, *Msx2*, *Wnt5a*, *Ctnnb1* and *Muc1* (**Fig 5D**). Overall, this analysis revealed that *Foxo1* ablation in the endometrium affects multiple signaling pathways with clear implications on key biological processes, including PGR signaling and inflammatory and growth factor pathways, which are required for proper uterine function during implantation.

### Mutually controlled balance of epithelial PGR and FOXO1 across the WOR

**Fig 6:**
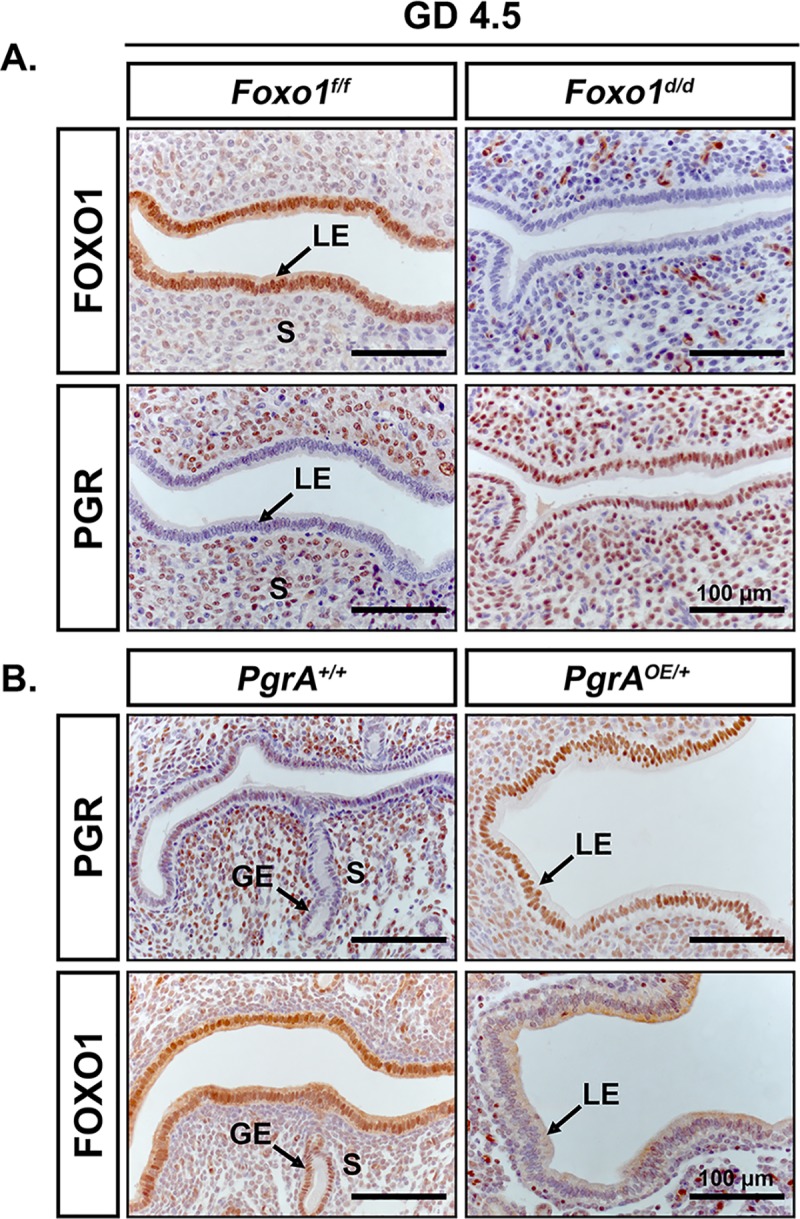
Mutually controlled balance of epithelial PGR and FOXO1 governs the WOR. Immunohistochemical staining for FOXO1 and PGR in the cross sections of *Foxo1*^*f/f*^ and *Foxo1*^*d/d*^ murine uteri at GD 4.5 (**A**), as well as, PGR and FOXO1 in the cross sections of *PgrA*^*+/+*^ and *PgrA*^*OE/+*^ murine uteri at GD 4.5 (**B**). LE, luminal epithelium; S, stroma. Scale bar, 100 μm.

Transcriptomic analysis of the *Foxo1*^*d/d*^ mouse uterus at day 4.5 of pregnancy showed activation of the PGR pathway. This loss of PGR expression has been proposed to play a defining role in the regulation of receptivity. Recently, we demonstrated that maintenance of PGR expression through the WOR inhibits embryo implantation blocks embryo implantation. [32]. The transcriptomic analysis would indicate that PGR signaling is maintained during the WOR. Analysis of the expression of PGR in the uterus of GD4.5 *Foxo1*^*f/f*^ and *Foxo1*^*d/d*^ mice showed higher levels of PGR in the LE than of *Foxo1*^*f/f*^ at GD 4.5 (**Fig 6A**) This is consistent with activation of PGR activity at PPD 4.5 by upstream regulator analyses of RNA-seq datasets (**Fig 5C**). Meanwhile, studies have previously shown that sustained progestin stimulation is required to maintain cytoplasmic sequestration of FOXO1 in decidualized human endometrial stromal cells [14, 18]. We observed that FOXO1 is predominantly cytoplasmic in the epithelium when PGR is present in the preimplantation phase, but becomes nuclear with downregulation of PGR during the WOR. These observations led us to hypothesize that downregulation of PGR may be necessary for epithelial FOXO1 localization and/or expression. Female mice with constitutive expression of PGR (*Wnt7a*^*Cre*^*PRA*^*LsL/+*^, i.e., *PgrA*^*OE/+*^) maintain epithelial expression of PGR-A across the WOR (**Fig 6B, top panel**) and exhibit infertility with defects in embryo attachment and stromal decidualization [32]. IHC analysis of the uteri of these mice showed a mutually controlled balance of FOXO1 and PGR, in which a much lower level of FOXO1 remained in the nucleus of epithelial cells of *PgrA*^*OE/+*^ than in the ones of the control group (*Wnt7a*^*Cre*^, i.e., *PgrA*^+/+^) at GD 4.5 (**Fig 6B, bottom panel**). Collectively, these observations indicate that spatiotemporal expression and localization of FOXO1 and PGR in the endometrial epithelium requires mutual regulation for successful implantation during pregnancy.

### Conserved FOXO1 and PGR balance in endometrial receptivity in humans

**Fig 7:**
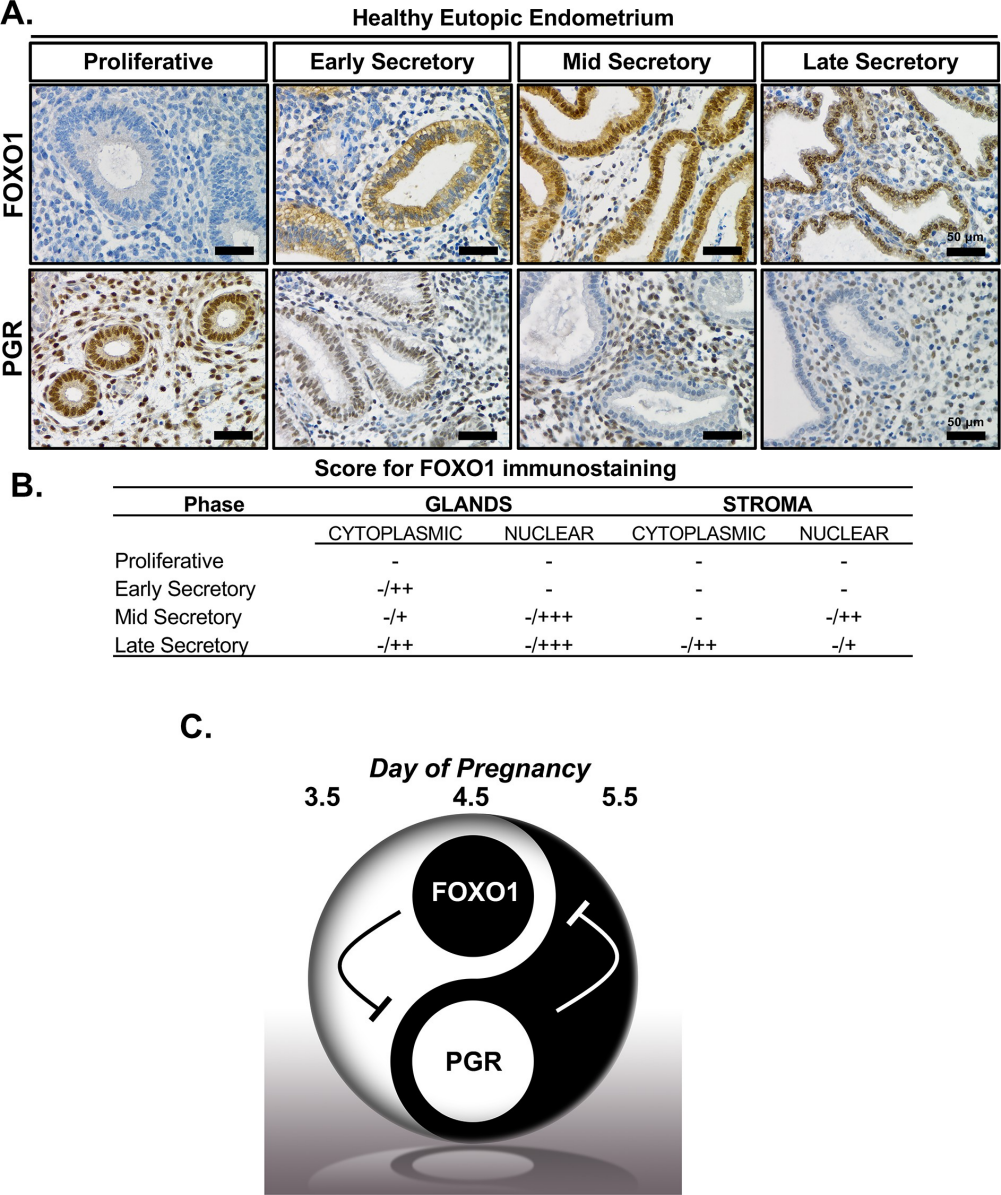
Conserved FOXO1 and PGR balance in endometrial receptivity in human. **(A)** Immunohistochemical staining for FOXO1 and PGR on endometrial sections from the functional layer of the endometrium in the proliferative, early secretory, mid-secretory, and late secretory phase of the menstrual cycle (*n* = 5). Scale bar, 50 μm. **(B)** Score for the compartmental and sub-cellular FOXO1 immunostaining across the menstrual cycle. Intensity indicated as follows: -, negative; +, weak; ++ moderate; +++, high. The slash is meant to show range given the variation in the samples (*n* = 5) **(C)** Proposed model for the temporal downregulation of *Pgr* in the LE of the endometrium by FOXO1 across the WOR.

To investigate the role of FOXO1 in human uterine receptivity, we analyzed its expression pattern along with the expression of PGR in endometrial tissue across the menstrual cycle of normal-ovulatory women. Endometrial tissue was staged according to urinary detection of luteinizing hormone surge and categorized as proliferative, early secretory, mid-secretory or late secretory phase. This spans the non-receptive stages, proliferative early and late secretory stages, and the receptive, mid secretory state in humans. Staining for FOXO1 was low or not detected in samples from the proliferative phase, and staining was positive for FOXO1 in the cytoplasm of the early-secretory phase. During the mid- and late secretory phase, FOXO1 staining was observed in the nucleus of the endometrial epithelium (**Fig 7A, top panels**). Blind scoring of the staining was performed independently and reported for FOXO1 in **Fig 7B.** The regulation of epithelial PGR expression appears to be conserved across the menstrual cycle in humans where expression of PGR is observed in the endometrial stroma and epithelium during the proliferative phase. Transition into the secretory phase involves a progressive down regulation of PGR expression in the endometrial epithelium (**Fig 7A, bottom panels**). These results indicate the expression pattern of FOXO1 and PGR, relative to the WOR, and is a feature that is conserved in mice and humans.

## Discussion

An intimate interaction between the implantation-competent blastocyst and a receptive uterus are required for successful embryo implantation. The uterine conditions required for this interaction are primarily regulated by progesterone via PGR-mediated transcriptional activity. PGR interacts with several factors in the transcriptional regulation of molecules required for endometrial receptivity, among these factors is FOXO1. Several studies have extensively characterized the importance of the PGR-FOXO1 interaction during the *in vitro* decidualization of human endometrial stromal cells [13, 14, 18, 33]. Until now, it remained unclear how FOXO1 regulates receptivity *in vivo*. In this study, we defined the expression of FOXO1 in endometrium across the reproductive cycle in humans and in mice. In order to evaluate the requirement and function of FOXO1 *in vivo*, a conditional loss-of-function FOXO1 mouse model was used to identify the FOXO1-dependent spatiotemporal expression of novel mediators of the blastocyst-maternal crosstalk.

Our immunohistochemical staining results indicate that FOXO1 and PGR exhibit a mutually exclusive expression pattern in the nucleus of endometrial epithelium across the female reproductive cycle in both humans and mice. At the WOR, mid-secretory phase in humans or GD 4.5 in mice, epithelial expression of PGR is down regulated, while expression of FOXO1 appears to exhibit a cytoplasmic to nuclear translocation. This observation is interesting because it had long been proposed that the relative nuclear/cytoplasmic location of these transcription factors can define the differentiation or survival fate of decidual cells [34]. Further, it had been demonstrated that continuous progestin stimulation is required to maintain FOXO1 sequestered in the cytoplasm for survival of decidual cells [33]. Here, we provide novel *in vivo* evidence for the FOXO1 translocation in the epithelial compartment of the endometrium through the WOR. The nuclear presence of FOXO1 coincides with the downregulation of PGR in that same compartment, indicating a potential mutually exclusive mechanism of regulation between FOXO1 and PGR in the uterine epithelium during the WOR. This is strongly supported by further evidence that *Foxo1* ablation retained PGR in the nuclei of uterine epithelia, and *PGR* overexpression inhibits nuclear FOXO1 during the WOR. Previous studies have suggested that PGR may inhibit PTEN, leading to enhanced activity of PI3K/Akt signaling pathway, and further inactivation of FOXO1 by phosphorylation and sequestration in the cytoplasm [34, 35].

Following this line of inquiry, we determined that *Foxo1* conditional knockout mice progress through pseudopregnancy without down-regulating the expression of the PGR in the epithelium. This evidence suggests a potential role of FOXO1 in the timely downregulation of epithelial PGR. Our transcriptomic data supports this hypothesis by identifying activation of PGR activity in the absence of FOXO1 in the murine uterine LE at GD 4.5. Based on RT-qPCR analyses, the retained PGR signaling was substantiated by increased mRNA levels of PGR responsive genes, such as *Pgr*, *Ihh*, *Msx1* and *Msx2*. Thus, it is possible to postulate that FOXO1 is involved in the downregulation of epithelial PGR, which accounted for the infertility in mice. Collectively, we propose that FOXO1 and PGR may be involved in a mutually regulatory relationship in the endometrial epithelium at the WOR, which was substantiated later by sequestration of FOXO1 in the cytoplasm in response to constitutive expression of PGR in the nuclei of endometrial epithelium during the time of implantation in mice.

This model used the Pgr^Cre^ mouse to ablate Foxo1 in all compartments of the uterus, as well as, the pituitary ovary axis. We demonstrated that the ovaries were able to ovulate and produce progesterone to maintain pregnancy. However, since we ablated Foxo1 in both the epithelial and stromal compartments of the uterus from birth, we cannot conclude that the resulting infertile phenotype is due solely to ablation of *Foxo1* in the uterine epithelium or due to a developmental defect. Although we did not see a reduction in the either the number of glands or the expression of FOXA2, we did see altered uterine epithelial cell proliferation and a delayed induction of LIF expression. However, the delayed in LIF expression was not the sole cause of the embryo implantation defect since rLIF could not rescue the phenotype. Also in the analysis of the growth of the uterus in an artificial decidual model, we observed a decrease in mass of the deciduoma at DD5 **(S3B Fig)**. This could indicate a developmental defect as well as a role for stroma expressed FOXO1 in the fertility phenotype. To address these issues of timing and the compartmental role of *Foxo1* in female fertility we crossed the *Foxo1*^*f/f*^ mouse to the *Ltf*^*Cre*^ mouse, which ablates genes specifically in the uterine epithelium starting at 90 days of age.[36]. The results of that breeding trial is shown in **S5 Fig.** The *Ltf*^*Cre*^*Foxo1*^*f/f*^ mice were sterile. Although this does rule out a developmental defect it does not rule out a role for stroma FOXO1 in the infertile phenotype. However, this analysis does demonstrate that epithelial expressed FOXO1 is critical for mouse fertility.

Previous studies had demonstrated FOXO1 functions as a transcriptional co-regulator of PGR for the differentiation of the endometrial stroma [13, 14]. It had been demonstrated that continuous progestin stimulation is required to maintain FOXO1 sequestered in the cytoplasm for survival of decidual cells [33]. However, it remained unknown if this interaction would be required for decidualization *in vivo*. To follow this line of evidence, we evaluated the ability of the *Foxo1*^*d/d*^ uteri to differentiate in response to a well-defined hormonal regimen for the artificial induction of decidualization. From this assay, we were able to determine that FOXO1 is not required to initialize the differentiation of the stroma, as the stimulated horns from both the control and conditional knockout mice exhibited a comparable increase in biomass at DD2. Interestingly, the decidual response at DD 5 in this artificial scheme was attenuated. The differences of the roles of FOXO1 in the mouse model and the decidualization of human endometrial stroma cells *in vitro* could be due to a stronger dependence of stroma cells on FOXO1 when cultured *in vitro* or species differences. Unlike the mouse, human stroma cells undergo a decidual reaction spontaneously, independent of embryo implantation. This spontaneous decidualization may be more dependent upon the action of FOXO1. However, there is conservation in the relationship of FOXO1 and PGR with respect to the cellular localization between the mouse and human in the endometrial compartments.

We observed by immunohistochemistry that decidual cells contain predominantly cytoplasmic signal for FOXO1. It is noteworthy to mention that PGR expression in the stroma is very robust during decidualization and further reiterates the mutually exclusive presence of PGR and FOXO1 in the endometrium. The cytoplasmic sequestration of FOXO1 in the mouse decidua indicates that nuclear FOXO1 may not play a critical role in the initiation of decidualization in the mouse model, but may play a role in secondary/late decidualization. Indeed, we observed a slight defect in biomass growth in the late decidual timepoint at DD5. We had previously demonstrated that conditional ablation of the wingless-related MMTV integration site 4 (*Wnt4*) using the *Pgr*^*cre/+*^ *Wnt4*^*f/f*^ mouse model results in uterine infertility and mislocalization of FOXO1 in artificially decidualized uterine horns [19]. The mislocalization of FOXO1 to the decidual cell nuclei was proposed to promote premature decidual cell apoptosis [19]. Collectively, this evidence suggests that FOXO1 may have a minimal role in the initiation of decidualization *in vivo*, but its sequestration in the cytoplasm of decidual cells is required for survival of the decidua. The sequestration of FOXO1 in the cytoplasm to prevent apoptosis could be conserved in the mouse uterine epithelium. The nuclear localization of FOXO1 in the uterine epithelium during the WOR could prepare the uterine epithelial cells for entosis and the ensuing apoptosis to allow embryo invasion.

The most striking finding from our evaluation of the reproductive phenotype of the *Foxo1*^*d/d*^ mice was that the decidualization reaction was initiated at sites of blastocyst apposition in the absence of trophoblastic penetration of the LE. This evidence indicated that the epithelium was competent to transmit the stimulus for decidualization to the stroma. The trophoblastic cells of the developing embryo need to breach the LE barrier as a requirement to establish the fetal-maternal vascular interphase. Their inability to do so in the *Foxo1*^*d/d*^ mice indicated that endometrial FOXO1 was required for the temporal permeability of the LE. Penetration of the uterine epithelium by polytene trophoblast cells during the invasion phase of implantation is aided by underlying decidual cells and required the timely removal of the epithelial barrier. In the normal murine endometrium, LE cells surrounding the blastocyst in the implantation chamber undergo a decrease in epithelial apical-basal polarity, as well as, a cell-shape transition from a columnar to cuboidal configuration approaching implantation, disappearing approximately 30 hours after the initiation of attachment [37]. This timely orchestrated transition of cell polarity is critical for blastocyst adhesion and invasion, thereby successful establishment of pregnancy [1]. Interestingly, the LE cells in the implantation chamber of *Foxo1*^*d/d*^ mice remained structurally intact at GD 5.5, evidenced by the strong and sharp staining of CDH1 at the apicolateral border of the LE, suggesting the greater apical basal polarity in *Foxo1*^*d/d*^ uteri during the implantation. MUC1, a well-known glycoprotein that serves as the cell-surface barrier to embryo implantation [38], maintained a high level in the apical membrane of LE in *Foxo1*^*d/d*^ uteri, indicating the abnormality of LE differentiation during the implantation. Meanwhile, the uterine LE (particularly the IS) during the receptive phase are devoid of signal from CLDN1, a tight junction protein that could alter the selective permeability of solutes across the LE, i.e., disturbing the transport of water, ions, and histotroph into the lumen [39]. The strong expression of CLDN1 in apicolateral membrane of the LE in *Foxo1*^*d/d*^ uteri suggested the critical role of FOXO1 in control of solute transport into lumen via LE depolarization. The absence of junction proteins GJB2 and TJP1 at the utero-embryonic interface in *Foxo1*^*d/d*^ mice suggested failure of embryo invasion via retained LE polarity. It subsequently disrupted intricate and precise communication between the blastocyst and the uterus, thereby causing embryonic death and infertility. Collectively, FOXO1 is required to accommodate embryo invasion by depolarizing and disintegrating the LE at the implantation chamber during implantation.

The mechanisms of LE clearance at the site of attachment are species-specific and are poorly characterized in the human due to ethical constraints. Interestingly, the apoptotic LE in the murine uterus is phagocytosed by the adjacent trophoblast [40]. Recently, it was demonstrated that during implantation, the LE cells in direct contact with the blastocyst are endocytosed by trophoblast cells in a process known as entosis [5]. This process initiates at GD 4.5 with the target cell undergoing a nonapoptotic cell death program and subsequent internalization by another live cell, leading to a transient state in which a live cell is contained within the host cell. Within 24 hours after this process initiates, apoptosis occurs in the AM extension of LE [41]. This process is driven by a Rho-dependent process requiring activity of the small GTPase, ROCK1 [5]. However, the extracellular stimulus and membrane component involved in triggering this intracellular signaling cascade resulting in the Rho-dependent cytoskeleton remodeling remains undefined. Our transcriptomic dataset further implicates FOXO1 in activation of cell invasion, molecular transport, apoptosis and the CTNNB1 signaling pathway, as well as, inhibition of PGR signaling in the LE during the time of implantation. However, further investigation will be required to evaluate the potential role of FOXO1 in the mechanism of entosis.

Targets of PGR, *Msx1*, *Msx2* and *Ihh* remained at higher levels in LE of *Foxo1*^*d/d*^ uteri, suggesting the inhibitory role of FOXO1 on PGR activity during implantation. On the other hand, WNT5A can induce cell polarity by promoting CDH1/CTNNB1 complex formation [42, 43] and was negatively regulated by *Msx1* and *Msx2* [37]. In this study, however, increase in *Wnt5a* in the context of higher *Msx1* and *Msx2* levels in LE of *Foxo1*^*d/d*^ uteri suggested that FOXO1 may regulate *Wnt5a* via direct and dominant inhibition. Notably, others have shown that gain-of-function overexpression of *Wnt5a* results in a subfertility phenotype associated with defects in LE organization, crypt formation, and embryo positioning [44].

In this study, we determined that ablation of FOXO1 results in infertility due to a uterine defect associated with inability of the blastocyst to invade the endometrial epithelium following an incomplete attachment reaction on the surface of the endometrium. The failure of embryo invasion was further identified as a consequence of defects in LE polarization and the ability of the uterine epithelium to be removed by trophoblast invasion, thereby disrupting intricate and precise communication between the blastocyst and the uterus. To elucidate the molecular mechanism for this implantation defect, we defined the FOXO1-dependent transcriptome at the WOR in the murine endometrium. This analysis determined that FOXO1 regulates the expression of multiple components of signaling pathways that establish the receptive phase including activation of cell invasion, molecular transport, apoptosis, CTNNB1 signaling pathway, as well as, inhibition of PGR signaling in the murine endometrial epithelium. The defective expression of molecules known to be required for fetal-maternal crosstalk in the *Foxo1* conditional knockout indicates that FOXO1 plays a major role in defining the WOR for embryo implantation. Particularly, the spatiotemporal expression and localization of FOXO1 and PGR in the endometrial epithelium in both human and mice, known as Yin-and-Yang balance (**Fig 7C**), are required for successful implantation during pregnancy. Collectively, these data identify FOXO1 as a marker and regulator of endometrial receptivity for the establishment of pregnancy.

## Materials and methods

### Ethics statement

This study was carried out in accordance with federal regulations governing human subjects research. All procedures were approved by the following ethics committees: Institutional Review Board/Committee-A (IRB) of Greenville Health System under IRB file # Pro00000993 and Pro00013885 and the University of Capel Hill at North Carolina IRB under file #: 05–1757. Written informed consent was obtained from all patients before their participation in this study.

All animal studies were conducted in accordance with the Guide for the Care and Use of Laboratory Animals published by the National Institutes of Health and animal protocols and approved by the Institutional Animal Care and Use Committee (IACUC) of Baylor College of Medicine under protocol number AN-4203 and by the Animal Care and Use Committee of National Institute of Environmental Health Sciences protocol numbers 2015–0012 and 2015–0023 Mice were euthanized by cervical dislocation following anesthesia. Mice undergoing euthanasia were anaesthetized with 100mg/kg sodium pentobarbital given I.P., 0.1ml/10 grams. Mice received irradiated Teklad global soy protein-free extruded rodent diet (Harlan Laboratories, Inc., Indianapolis, IN) and fresh water ad libitum.

### Generation of transgenic mice

Generation of the conditional allele targeting the second major coding exon of *Foxo1* (encoding the C-terminal half of the full-length protein) was previously described [20]. Floxed Foxo1 (*Foxo1*^*f/f*^) mice were crossed with the *Pgr*^*Cre*^ mouse model [16] to generate conditional knockout animals, i.e., *Foxo1*^*d/d*^. Floxed Foxa2 (*Foxa2*^*f/f*^) mice were crossed with the *Pgr*^*Cre*^ mouse model to generate conditional knockout animals, i.e., *Foxa2*^*d/d*^. Generation of the conditional *mPgrA*^*LsL*^ allele was previously described [32]. Briefly, the transgenic mice were generated by placing the PGR-A cDNA in a vector containing the hybrid chicken β-actin ubiquitous promoter (CAGGS) and a LoxP-STOP-LoxP (LsL) cassette inserted into the murine ROSA26 locus. Expression of mouse PGR-A (*PgrA*^*LsL/+*^) was driven in the whole uterus by crossing this mouse line with the *Wnt7a*^*Cre*^ (epithelial-Cre) line. Presence of both alleles drives recombination of the LoxP sites and excision of the STOP cassette, driving constitutive expression of PGR-A across the uterus. In addition, *Foxo1*^*f/f*^ mice were crossed with the *Ltf*^*iCre*^ mouse model to generate endometrial epithelial knockout mice (*Foxo1*^*ed/ed*^).

### Fertility trial

Conditional knockout and control littermate females at approximately 6 weeks of age were housed individually and continuously with males of the B6D2F1 strain. Mating was confirmed by presence of vaginal plugs. Fertility was assessed by monitoring frequency of litters and litter sizes for a six-month period.

### Implantation and pseudopregnancy

Blastocyst implantation was measured by mating 8-week-old females with wild-type males. The morning a vaginal plug was observed was considered 0.5 days post coitus (GD 0.5). Mice were sacrificed at gestational day (GD) 4.5, 5.5, 6.5, 7.5, 8.5 and 9.5. Uteri were excised, imaged, and IS diameter was measured using a Vernier caliper. Whole uteri were fixed in 4% v/v electron microscopy grade paraformaldehyde (PFA) in phosphate buffered saline (PBS) for histology on sagittal sections. Histological analysis of serial sagittal sections of both uterine horns at GD 5 was conducted to determine the number of embryos attached versus unattached to the uterine epithelium. Analysis of uterine gene expression during pseudopregnancy was accomplished by mating 8-week-old female mice with vasectomized male mice. The presence of the postcoital vaginal plug was designated pseudopregnant day (PPD) 0.5. The mice were sacrificed on PPD 0.5, 1.5, 2.5, 3.5, 4.5 and 5.5.

### Mouse surgeries and exogenous hormone treatments

All surgeries were performed using avertin anesthesia (2.5% (v/v) solution, 0.02 ml/g body weight) and postoperative pain was alleviated with the analgesic, ketoprofen (5–10 mg/kg of body weight). Ovariectomy was performed on 6-week-old female mice by exposing the reproductive tract via a dorsolateral incision. The ovaries were ligated with sterile absorbable suture and excised. The peritoneal opening was closed with absorbable suture and the skin closed with wound clips. Females were rested for 2 weeks to deplete endogenous hormones. Hormones were dissolved in sesame oil and administered via 0.1 ml subcutaneous injections.

For preimplantation gene expression studies, mice were given 3 daily injections of 100 ng estradiol followed with 2 days of rest and then a combination of 6.7 ng of estradiol with 1 mg of progesterone for 2 days. Mice were sacrificed 6 hours after the final injection. Uterine horns were flash frozen for RNA analysis or fixed in 4% v/v PFA in PBS for histology. This hormone strategy was utilized to mimic preimplantation day 3.5. For the artificial induction of decidualization, females were primed with 3 daily subcutaneous injections of 100 ng estradiol and then rested for 2 days. Mice subsequently received 3 daily injections of 1 mg progesterone and 6.7 ng estradiol per mouse subcutaneously. One uterine horn was exposed by a dorsolateral incision and stimulated by instillation of sesame oil into the uterine lumen 6 hours after the third injection of progesterone and estradiol. The reproductive tract was returned to the peritoneum and the opening closed as described above. Mice continued to receive daily injections of progesterone and estradiol until sacrificed at day 2 (decidual day 2, DD 2) or day 5 (DD5) post surgery.

### LIF rescue assay

Adult females (*Foxo1*^*f/f*^, *Foxo1*^*d/d*^, and *Foxa2*^*d/d*^) were mated overnight with wild-type males. Females were separated from males the following morning. Presence of a vaginal plug was considered 0.5 day post coitus (GD 0.5). Female mice received two intraperitoneal (i.p.) injections (one at 1000 h and one at 1800 h) of vehicle or 10 μg recombinant mouse LIF on GD 3.5. Embryo implantations were evaluated on GD 5.5 by eosin and hematoxylin staining.

### Blastocyst flush

Adult females (*Foxo1*^*f/f*^ and *Foxo1*^*d/d*^) were mated overnight with wild-type males. Females were separated from males the following morning. Presence of a vaginal plug was considered 0.5 day post coitus (GD 0.5). Uterine horns were harvested at GD 3.5. Blastocysts were recovered by phosphate-buffered saline and the numbers were counted under a bright-field microscope.

### Histological and immunohistochemical staining

At the time of sacrifice, a mid-portion of the uterine horn was fixed in 4% v/v PFA in PBS overnight at room temperature and followed by three washes with 70% ethanol. Dehydrated tissues were embedded in paraffin and sectioned to 5 um thickness on glass slides. Slides were heated for 20 min at 60°C and cooled for 10 min. Sections were dehydrated in 3 washes of xylenes for 5 minutes each and hydrated in ethanol gradients (100%, 95% and 70%).

For eosin and hematoxylin staining, slides were incubated in hematoxylin for 3 minutes, washed with water and developed in a 10% lithium carbonate solution. Slides were subsequently incubated with eosin for 1 minute, washed and dehydrated in an ethanol gradient and finally in xylene washes. Slides were mounted with Permount and glass coverslips.

For immunohistochemical staining, hydrated sections were boiled with unmasking solution (Vector Laboratories Burlingame, CA) and washed three times with PBS. Endogenous peroxidases were quenched by incubating sections with 3% (v/v) hydrogen peroxide in methanol for 10 minutes in the dark. Sections were washed and blocked with 10% (v/v) normal goat serum in PBS. Sections were incubated over night at 4°C in a humidified chamber with primary antibodies (see **S4 Table**). Following washes in PBS, slides were incubated with anti-rabbit biotinylated secondary antibodies (5 μL/mL) for 1 hour at RT in humidified chambers. Secondary antibody was removed and slides were washed three times with PBS. VECTASTAIN Elite ABC Reagent and Vector DAB peroxidase substrate kits were utilized for development of staining and optimized for each antibody condition (Vector Laboratories Burlingame, CA). Tissue was counterstained with hematoxylin and dehydrated before affixing coverslips. A semiquantitative grading system (H-score) was used to compare the immunohistochemical staining intensities. The H-score was calculated using the following equation: H-score = ∑ Pi (i), where i = intensity of staining with a value of 1, 2 or 3 (weak, moderate or strong, respectively) and Pi is the percentage of stained cells for each intensity, varying from 0 to 100%.

### *In situ* localization of Hbegf mRNA

RNAscope *in situ* hybridization (Advanced Cell Diagnostics, Newark, CA) was performed according to the manufacturer’s instructions using paraformaldehyde-fixed tissues and a mouse Hbegf probe (Catalog # 437601, Advanced Cell Diagnostics, Newark, CA). Following hybridization, slides were washed and probe binding visualized using the HD 2.5 Red Detection Kit (Catalog # 322350, Advanced Cell Diagnostics, Newark, CA). Sections were briefly counterstained with hematoxylin before dehydrating and affixing coverslips with Permount.

### Western blot analysis of protein expression

Tissue was homogenized in 600 μL of protein lysis buffer (10 mM Tris, pH 7.4, 150 mM NaCl, 2.5 mM EDTA and NP-40) supplemented with protease inhibitor cocktail (cOmplete Mini, EDTA-free, REF 11-836-170-001, Roche Diagnostics, Mannheim, Germany) and phosphatase inhibitor cocktail (PhosSTOP, REF 04-906-837-001, Roche Diagnostics, Mannheim, Germany) 6 times for 10 seconds and rested intermittently for 10 seconds on ice. Homogenates were centrifuged 10 minutes at 11,400 rpm in 4°C to pellet cellular debris. Supernatant was transferred to a new 1.5 mL centrifuge tube.

Denatured protein extracts (10 μg) per sample were loaded on a Bis-Tris NuPAGE 4–12% gel (REF NP0321BOX, Novex by Life Technologies, Carlsbad, CA) for electrophoresis separation. Proteins were transferred to polyvinylidene difluoride membranes, PVDF, (MilliporeSigma, Burlington, MA) in transfer buffer (25 mM Tris, 192 mM glycine, and 20% methanol) (Life Technologies, Carlsbad, CA). PVDF membranes were subsequently blocked with 5% (w/v) blotting grade non-fat milk (#170–6404, BIO-RAD, Hercules, CA) in PBS containing 0.1% tween 20 for 1 hour at room temperature. Membranes were probed with antibodies for FOXO1 (Cell Signaling, Danvers, MA, #2880), and β ACTIN (Sigma-Aldrich, St. Louis, MO, Catalog No. A5441) overnight at 4°C in 5% (w/v) blotting grade non-fat milk in PBS containing 0.1% (v/v) tween 20. Blotted membranes were subsequently washed three times with PBS containing 0.1% tween 20 and incubated 1 hour at room temperature with secondary antibody (anti-rabbit peroxidase and anti-mouse peroxidase, accordingly). Blots were washed three times with PBS containing 0.1% (v/v) tween 20 and an additional three times with PBS only. The Amersham ECL Western Blotting System (GE Healthcare, Chicago, IL) was utilized for the luminol-based detection of bands on film as per manufacturer’s instructions.

### RNA isolation and reverse transcription real-time polymerase chain reaction (RT-qPCR)

Uterine horns were dissected from anesthetized mice two weeks after each respective surgery and cervixes were discarded. Tissue was homogenized in 1 mL of TriZOL 6 times for 10 seconds and rested intermittently for 10 seconds on ice. Homogenates were centrifuged 10 minutes at 11,400 rpm in 4°C to pellet cellular debris. Supernatant was transferred to a new 1.5 mL centrifuge tube and mixed with 200 μL of chloroform by shaking the tubes. Samples were rested for 3 minutes at room temperature and subsequently centrifuged at max speed for 18 minutes at 4 ^o^C. Approximately 600 μL of the aqueous layer was transferred to a new tube and mixed with equal parts of 70% ethanol. This mix was filtered in columns from the RNAeasy Mini kit (QIAGEN, Germantown, MD). Columns were washed once with 700 μL of RWT buffer and three times with 500 μL of RPE buffer. RNA was eluted with RNase free water.

RNA was reverse transcribed into cDNA with Moloney Murine Leukemia Virus (M-MLV, Life Technologies, Carlsbad, CA) according to manufacturer’s recommendations. Expression levels of mRNA were determined by RT-qPCR on a QuantStudio 12K Flex Real-Time qPCR system (Life Technologies, Carlsbad, CA) using the TaqMan® Gene Expression Assay platform (Life Technology, Carlsbad, CA) or FastStart SYBR Green Master (Roche Diagnostics, Indianapolis, IN) with oligonucleotide primers synthesized by Sigma-Aldrich (St. Louis, MO). Gene expression was normalized to 18s rRNA by ΔΔCT method.

### RNA-seq

RNA was prepared for sequencing by the Genomic and RNA Profiling Core at Baylor College of Medicine, Houston, TX. Sample quality checks were performed using the NanoDrop spectrophotometer and Agilent Bioanalyzer 2100. Libraries were generated using the Illumina TruSeq RNA library preparation protocol. A double-stranded DNA library was created using 250 ng of total RNA, quantitated by picogreen, preparing the fragments for hybridization onto a flow cell. First, cDNA was created using the fragmented 3’ poly (A) selected portion of total RNA and random primers. Libraries were created from the cDNA by first blunt ending the fragments, attaching an adenosine to the 3’ end and finally ligating unique adapters to the ends. The ligated products were then amplified using 15 cycles of PCR. The resulting libraries were quantitated using the NanoDrop spectrophotometer and fragment size assessed with the Agilent 2100 Bioanalyzer. A qPCR assay was performed on the libraries to determine the concentration of adapter ligated fragments using the Applied Biosystems ViiA 7 Quantitative PCR instrument and a KAPA Library Quant Kit. All samples were pooled equimolar and requantified by qPCR, and reassessed on the Bioanalyzer. Using the pooled concentration from the qPCR assay, the library pool was loaded onto each of two rapid run flow cells at a concentration of 12 pM, for on-board cluster generation and sequencing on the HiSeq 2500 at a read length of 100 bp, paired-end.

Read mapping and gene differential expression analysis were performed by the Biostatistics and Analytics Group in the Dan L. Duncan Comprehensive Cancer Center Baylor College of medicine, Houston, TX. In order to increase the mappability, we trimmed 11 low-quality nucleotides from the 5’ ends of the reads. The resulting 90-nucleotide pair-ended reads were mapped to the mouse genome (UCSC mm10) using STAR with NCBI RefSeq genes as the reference. [45] In order to reduce possible PCR biases, we removed the read duplicates using picard tools (http://broadinstitute.github.io/picard/). HTseq was used to determine the number of reads falling in the known genes [5]. EdgeR was used to analyze the gene-based read counts to detect differentially expressed genes between the control and treatment groups. [46] The false discovery rate (FDR) of the differentially expressed genes was estimated using the Benjamini and Hochberg method. FDR < 0.05 was considered statistically significant. Files were deposited to GEO Datasets under accession number GSE72895.

### Data analysis

The differentially genes generated from RNA-seq data were analyzed using Ingenuity Pathway Analysis software (IPA, www.ingenuity.com) and Database for Annotation, Visualization, and Integrated Discovery (DAVID, https://david.abcc.ncifcrf.gov). The GraphPad Prism software was implemented for one-way analysis of variance (ANOVA), Tukey-Kramer multiple comparison test, and Student t-test analyses for RT-qPCR, litter production in the breeding trial, growth of the ISs over early pregnancy, and decidual wet weights. Hierarchal clustering heatmaps were generated using Partek Genomics Suite 6.6 software.

## Supporting information

S1 Fig Implantation sites of *Foxo1* ablated mice lack embryos. Females at 6 weeks of age were mated with fertile males. Presence of vaginal plug indicated postcoital day 0.5 (GD 0.5). **(A)** Gross morphology of uterine horns with visible ISs. Scale bar, 1 cm. **(B)** Quantification of IS diameter. Data are presented as means ± SEM. **, *P<*0.01; ***, *P*<0.001. **(C)** Eosin and hematoxylin staining was performed on transverse sections of individual ISs. The midportion of each attachment sites in *Foxo1*^*f/f*^ and *Foxo1*^*d/d*^ are shown for days 7.5, 8.5 and 9.5. *n* = 4.
(TIF)

S2 Fig Ablation of *Foxo1* interferes with the LIF production but hinders the embryo implantation in a LIF-independent fashion. **(A)** Quantification of the *Lif* gene in uteri from *Foxo1*^*f/f*^ and *Foxo1*^*d/d*^ mice (*n* = 6) at GD 3.5 and 4.5. Data are presented as means ± SEM. *, *P*<0.05. **(B)** Embryo implantations were observed by eosin and hematoxylin staining on GD 5.5 in vehicle-treated *Foxo1*^*f/f*^ mice (*n* = 5) and in LIF-replaced *Pgr*^*Cre/+*^*Foxa2*^*f/f*^ (*Foxa2*^*d/d*^, *n* = 4) mice but neither in vehicle-treated nor LIF-replaced *Foxo1*^*d/d*^ mice (*n* = 5). Scale bar, 100 μm.
(TIF)

S3 Fig Ablation of *Foxo1* results in minor defects in decidualization. Ovariectomized mice were treated with exogenous hormones and a deciduogenic stimulus was administered to the left uterine horn. **(A)** Gross uterine morphology of uteri (*n* = 4) at decidual days 2 (DD2) and 5 (DD5). Scale bar, 1 cm. **(B)** Wet weight ratio of decidual stimulated uterine horn relative to unstimulated horn. Data are presented as means ± SEM, *n* = 4. *, *P*<0.05. **(C)** Histological analysis of FOXO1 expression in stimulated uterine cross sections between *Foxo1*^*f/f*^ and *Foxo1*^*d/d*^ at DD2 (*n* = 4). Arrows indicate endometrial compartments as follows: LE, luminal epithelium, GE, glandular epithelium, and S, stroma. AM, antimesometrial pole; M, mesometrial pole. Scale bar, 100 μm. **(D)** Histological analysis of FOXO1 expression in stimulated uterine cross sections between *Foxo1*^*f/f*^ and *Foxo1*^*d/d*^ at DD5 (*n* = 4). Arrow indicates the luminal epithelium and grey triangle indicates the primary decidual zone, solid black arrows indicate secondary decidual zone. Scale bar, 50 μm.**(E)** Quantification of *Foxo1*, *Bmp2 and Wnt4* genes in both decidual and control uterine horn from *Foxo1*^*f/f*^ and *Foxo1*^*d/d*^ mice at DD5 (*n* = 4). Data are presented as means ± SEM, *n* = 4. *, *P*<0.05.
(TIF)

S4 Fig Ablation of *Foxo1* retains the polarity of uterine epithelium in GD 5 pregnant mice. (**A**) Immunohistochemical staining for Mucin-1 (MUC1), E-cadherin (CDH1), Connexin 26 (GJB2), ZO-1 (TJP1) and Claudin 1 (CLDN1) in the cross sections of both *Foxo1*^*f/f*^ and *Foxo1*^*d/d*^ murine uteri at GD 5.5. Scale bar, 100 μm. (**B**) Immunohistochemical staining for MUC1 in GD 5.5 uteri of *Foxo1*^*f/f*^ and *Foxo1*^*d/d*^ mice at low magnification. M, mesometrial pole; AM, antimesometrial pole; E, embryo; IS, implantation site; I-IS, inter-implantation site. Scale bar, 1 mm.
(TIF)

S5 Fig The number of pups born from *Foxo1^f/f^* and *Ltf^iCre/+^Foxo1^f/f^* (*Foxo1^ed/ed^*) female mice (n = 4) in 6-month breeding trial. Data are presented as means ± SEM. *, *P*<0.05.
(TIF)

S1 Table List of differentially expressed genes in Foxo1^d/d^ mouse uteri compared with Foxo1^f/f^ mouse uteri at PPD 4.5. (XLSX)

S2 Table Functional annotation of altered transcriptome in Foxo1^d/d^ mouse uteri at PPD 4.5. (XLSX)

S3 Table Functional annotation of altered transcriptome in Foxo1^d/d^ mouse uteri at PPD 4.5. (XLSX)

S4 Table Antibody information. (XLSX)
