# Supplementary figures and images for: FOXO1 regulates uterine epithelial integrity and progesterone receptor expression critical for embryo implantation
Source: PLoS Genet. 2018 Nov 19;14(11):e1007787. doi: 10.1371/journal.pgen.1007787 (PMC6277115; doi:10.1371/journal.pgen.1007787)

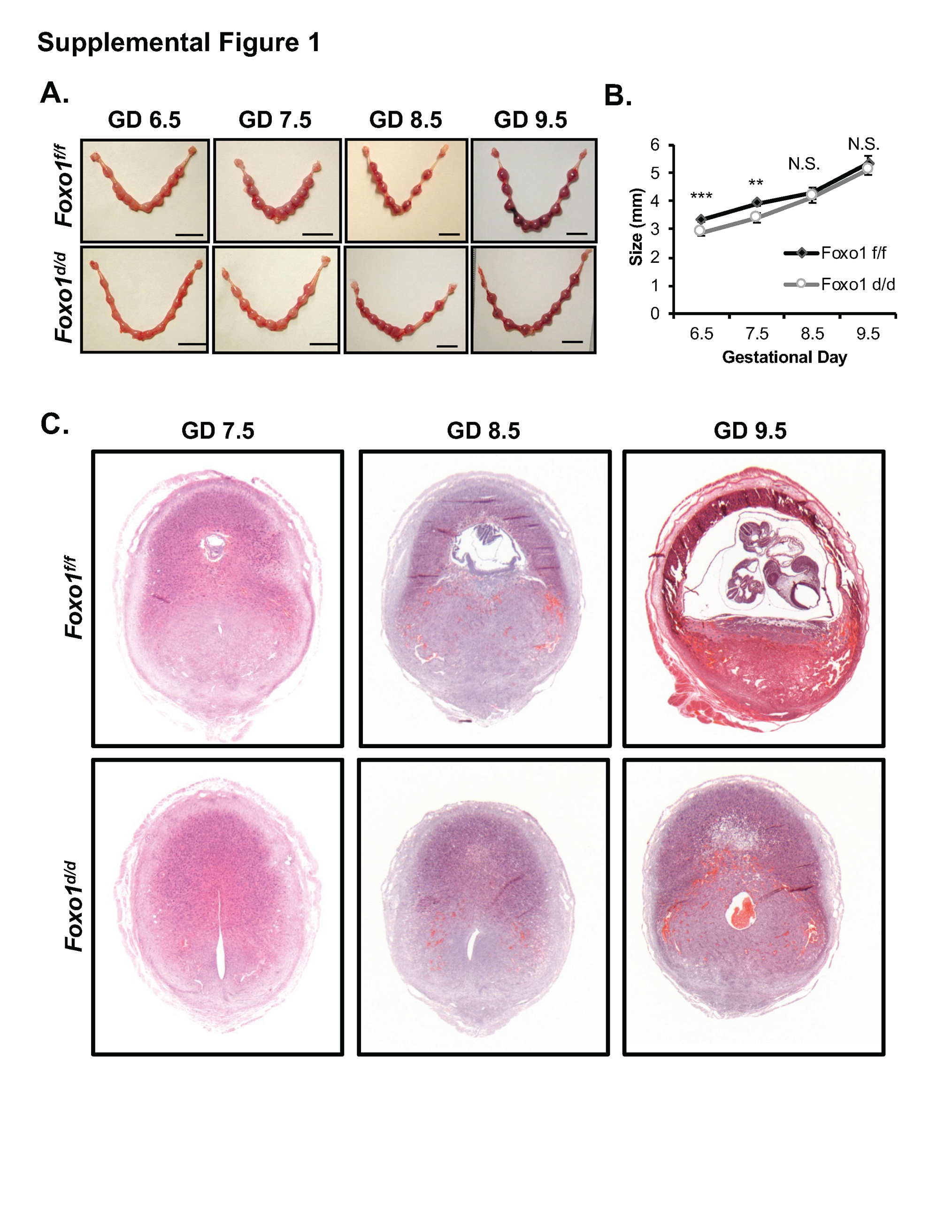

Supplement: S1 Fig — Females at 6 weeks of age were mated with fertile males. Presence of vaginal plug indicated postcoital day 0.5 (GD 0.5). (A) Gross morphology of uterine horns with visible ISs. Scale bar, 1 cm. (B) Quantification of IS diameter. Data are presented as means ± SEM. **, P<0.01; ***, P<0.001. (C) Eosin and hematoxylin staining was performed on transverse sections of individual ISs. The midportion of each attachment sites in Foxo1f/f and Foxo1d/d are shown for days 7.5, 8.5 and 9.5. n = 4. (TIF) [file pgen.1007787.s001.tif]

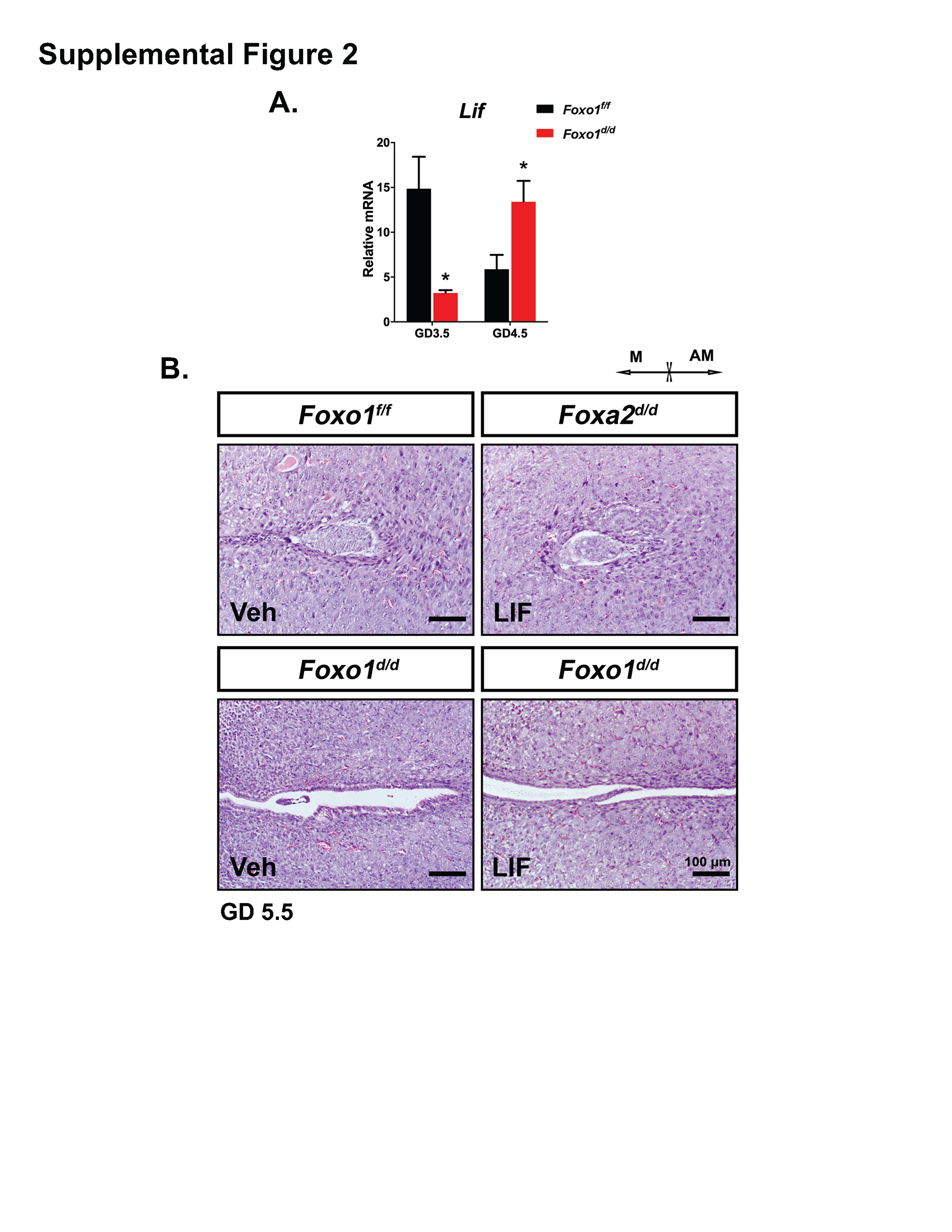

Supplement: S2 Fig — (A) Quantification of the Lif gene in uteri from Foxo1f/f and Foxo1d/d mice (n = 6) at GD 3.5 and 4.5. Data are presented as means ± SEM. *, P<0.05. (B) Embryo implantations were observed by eosin and hematoxylin staining on GD 5.5 in vehicle-treated Foxo1f/f mice (n = 5) and in LIF-replaced PgrCre/+Foxa2f/f (Foxa2d/d, n = 4) mice but neither in vehicle-treated nor LIF-replaced Foxo1d/d mice (n = 5). Scale bar, 100 μm. (TIF) [file pgen.1007787.s002.tif]

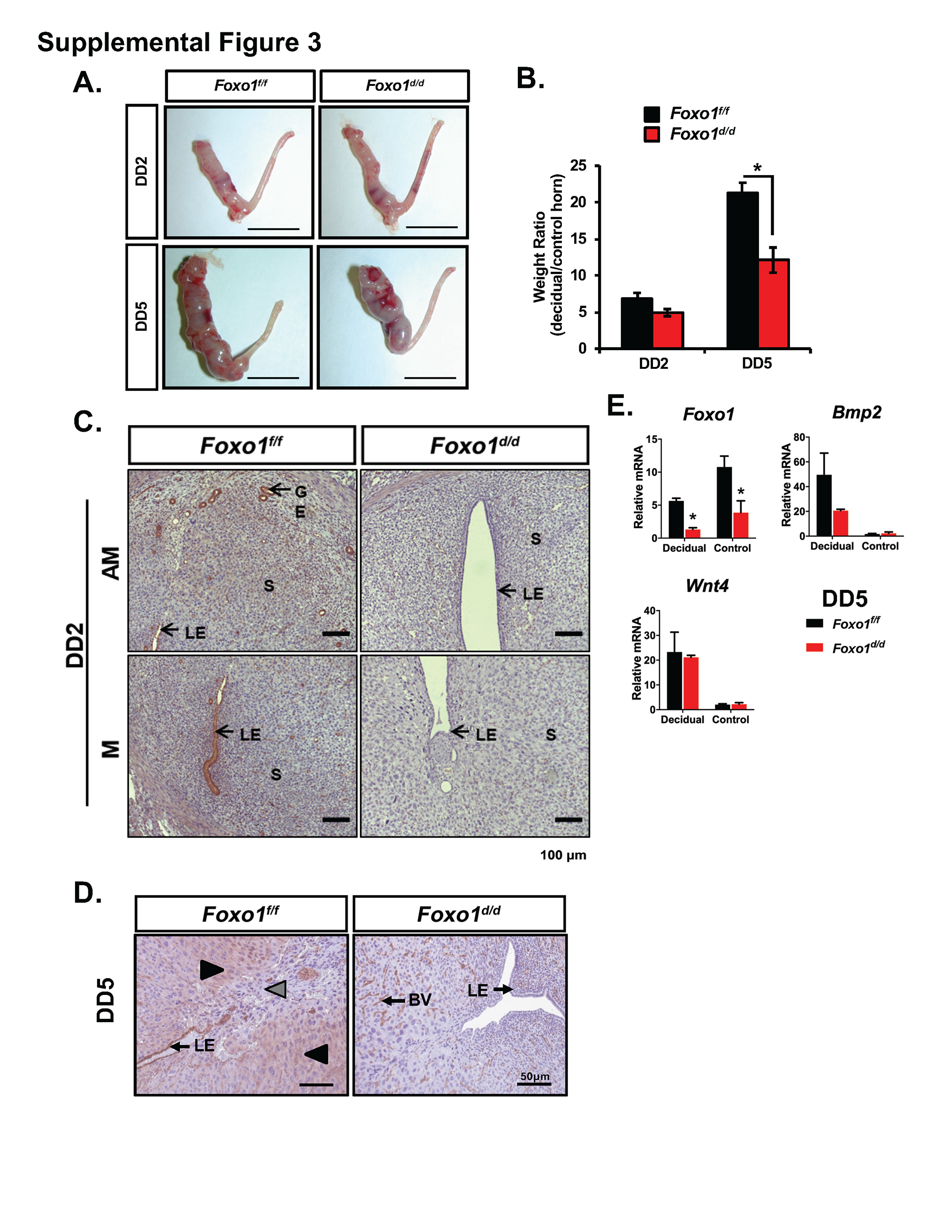

Supplement: S3 Fig — Ovariectomized mice were treated with exogenous hormones and a deciduogenic stimulus was administered to the left uterine horn. (A) Gross uterine morphology of uteri (n = 4) at decidual days 2 (DD2) and 5 (DD5). Scale bar, 1 cm. (B) Wet weight ratio of decidual stimulated uterine horn relative to unstimulated horn. Data are presented as means ± SEM, n = 4. *, P<0.05. (C) Histological analysis of FOXO1 expression in stimulated uterine cross sections between Foxo1f/f and Foxo1d/d at DD2 (n = 4). Arrows indicate endometrial compartments as follows: LE, luminal epithelium, GE, glandular epithelium, and S, stroma. AM, antimesometrial pole; M, mesometrial pole. Scale bar, 100 μm. (D) Histological analysis of FOXO1 expression in stimulated uterine cross sections between Foxo1f/f and Foxo1d/d at DD5 (n = 4). Arrow indicates the luminal epithelium and grey triangle indicates the primary decidual zone, solid black arrows indicate secondary decidual zone. Scale bar, 50 μm.(E) Quantification of Foxo1, Bmp2 and Wnt4 genes in both decidual and control uterine horn from Foxo1f/f and Foxo1d/d mice at DD5 (n = 4). Data are presented as means ± SEM, n = 4. *, P<0.05. (TIF) [file pgen.1007787.s003.tif]

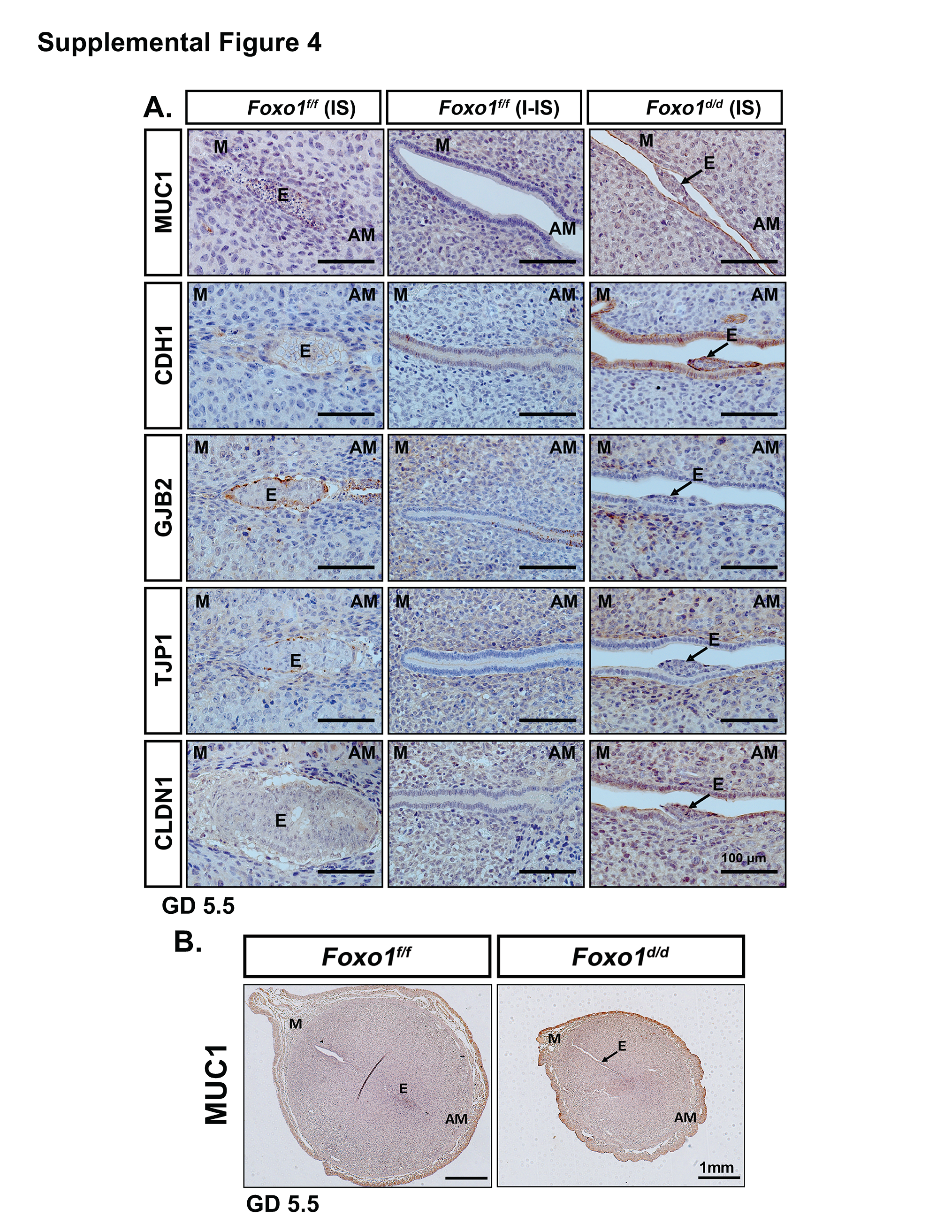

Supplement: S4 Fig — (A) Immunohistochemical staining for Mucin-1 (MUC1), E-cadherin (CDH1), Connexin 26 (GJB2), ZO-1 (TJP1) and Claudin 1 (CLDN1) in the cross sections of both Foxo1f/f and Foxo1d/d murine uteri at GD 5.5. Scale bar, 100 μm. (B) Immunohistochemical staining for MUC1 in GD 5.5 uteri of Foxo1f/f and Foxo1d/d mice at low magnification. M, mesometrial pole; AM, antimesometrial pole; E, embryo; IS, implantation site; I-IS, inter-implantation site. Scale bar, 1 mm. (TIF) [file pgen.1007787.s004.tif]

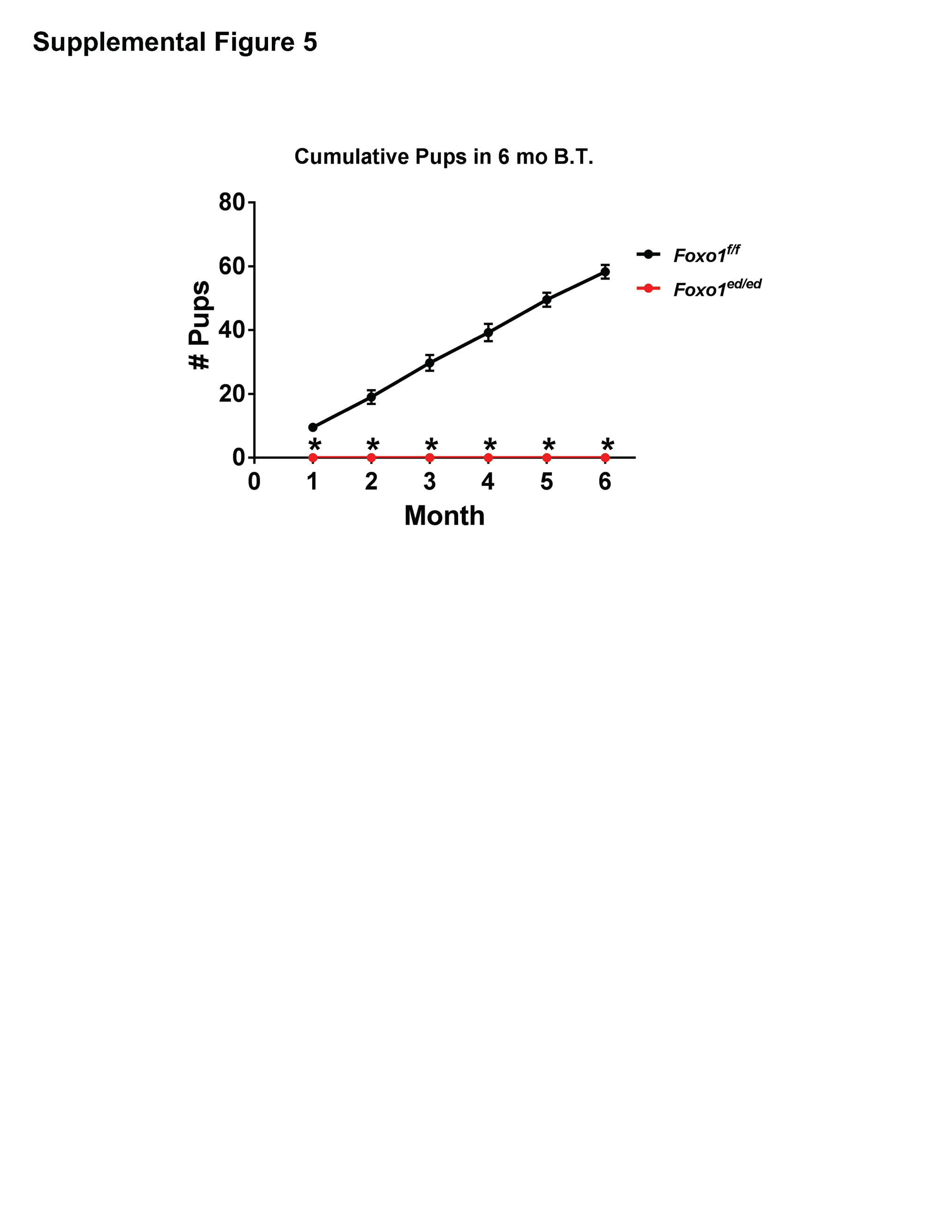

Supplement: S5 Fig — Data are presented as means ± SEM. *, P<0.05. (TIF) [file pgen.1007787.s005.tif]
